# Potential Relevance of Amazonian Diet Components in Parkinson’s Disease: An Integrative Review with Multivariate Analysis

**DOI:** 10.3390/nu18152472

**Published:** 2026-07-30

**Authors:** Maria Fernanda Manica-Cattani, Ivana Beatrice Mânica da Cruz, Euler Esteves Ribeiro, Raquel de Souza Praia, Cristina Maranghello, Ivo Emilio Jung, Vitória Farina Azzolin, Railla da Silva Maia, Marco Aurélio Echart Montano, Vanusa Nascimento, Eduardo Vélez Martin, Verônica Farina Azzolin

**Affiliations:** 1 Centro Universitário da Serra Gaúcha (FSG), Caxias do Sul 95020-371, RS, Brazil; fernanda18cattani@gmail.com; 2 Centro de Ensino, Pesquisa e Desenvolvimento Tecnológico GERONTEC, Fundação Universidade Aberta da Terceira Idade, FUnATI, Manaus 69029-040, AM, Brazil; 3 Programa de Pós-Graduação em Imunologia Básica e Aplicada, Universidade Federal do Amazonas, Manaus 69080-900, AM, Brazil; 4 Programa de Pós-Graduação em Gerontologia, Universidade Federal de Santa Maria, Santa Maria 97105-900, RS, Brazil; 5 Programa de Pós-Graduação em Ciências do Envelhecimento, Universidade São Judas Tadeu, São Paulo 03166-000, SP, Brazil; 6 Programa de Pós-Graduação em Biotecnologia, Universidade Federal do Amazonas, Manaus 69080-900, AM, Brazil

**Keywords:** Amazonian Diet, Parkinson’s disease, mitochondrial dysfunction, neuroinflammation, bioactive compounds, dietary patterns, gut–brain axis

## Abstract

Dietary patterns increasingly influence research on neurodegenerative diseases, with attention shifting from isolated nutrients to integrative nutritional models. The Amazonian Diet, a biodiversity-based dietary pattern rich in native fruits, seeds, freshwater fish, and cassava-derived foods, is naturally enriched in bioactive compounds including polyphenols, anthocyanins, carotenoids, methylxanthines, selenium, vitamins, and unsaturated fatty acids. This review aimed to investigate the potential relevance of key foods derived from the Amazonian Diet to Parkinson’s disease (PD) by integrating compositional nutritional analysis, multivariate analytical approaches, and mechanistic evidence synthesis. In Stage 1, the composition of 36 Amazonian foods was analyzed using TBCA and FAO data, followed by hierarchical clustering analysis (Ward’s linkage, Euclidean distance). Distinct compositional patterns were identified, highlighting foods with high bioactive diversity, relevant lipid composition, and dietary fiber. In Stage 2, an integrative literature review (PubMed/MEDLINE, SciELO) of in vitro, in vivo, observational, and clinical studies suggested that açaí berry, guaraná, cocoa/cacao, camu-camu, and Brazil nuts contain nutrients and bioactive compounds that intersect with biological pathways implicated in PD, including oxidative stress, mitochondrial dysfunction, and neuroinflammation. However, this review does not evaluate the effects of the Amazonian Diet on PD incidence, progression, symptoms, levodopa response, or biomarkers.

## 1. Introduction

Parkinson’s disease (PD) is a progressive neurodegenerative disorder characterized by the selective loss of dopaminergic neurons in the substantia nigra pars compacta, resulting in cardinal motor symptoms such as resting tremor, rigidity, and bradykinesia, as well as a wide range of non-motor manifestations, including gastrointestinal dysfunction, sleep disturbances, mood disorders, and cognitive impairment [1]. While the etiology of PD is multifactorial, aging is widely recognized as the most significant risk factor for the disorder [2]. PD is the second most prevalent neurodegenerative disease worldwide, affecting approximately 1% of individuals over 60 years of age, and represents a growing public health burden, particularly in low- and middle-income countries, which account for approximately 44% of all individuals with PD. It can also affect individuals under the age of 50 [3,4].

In Brazil, studies derived from the governmental health system databank (DATASUS) have reported regional differences in Parkinson’s disease records, with fewer cases documented in the Northern region than in the South and Southeast regions [5,6]. Such variation is likely influenced by multiple demographic, socioeconomic, environmental, and healthcare-related factors, including age structure, diagnostic access, healthcare utilization, survival, migration, occupational and environmental exposures, pesticide exposure, and differences in case ascertainment. These regional patterns highlight the complexity of the epidemiology of Parkinson’s disease in Brazil and underscore the importance of investigating a broad range of environmental and lifestyle factors, including dietary patterns, that may contribute to disease risk and progression [3].

Among environmental determinants, diet has increasingly been investigated as a potentially modifiable factor influencing neurodegenerative processes. Evidence suggests that dietary patterns rich in fruits, vegetables, and fish may exert protective effects against neurodegeneration through mechanisms involving antioxidant activity, anti-inflammatory effects, modulation of mitochondrial function, and regulation of neuronal survival pathways. For example, adherence to dietary patterns such as the Mediterranean Diet has been associated with a lower risk of PD and a later age at disease onset in observational studies [7]. These effects are thought to be mediated by the high intake of plant-derived bioactive compounds, unsaturated fatty acids, and other nutrients capable of modulating oxidative stress, neuroinflammation, and mitochondrial dysfunction—key processes involved in PD pathophysiology.

Evidence from Mediterranean, DASH (Dietary Approaches to Stop Hypertension), and MIND (Mediterranean-DASH Intervention for Neurodegenerative Delay) dietary patterns supports the concept that whole dietary patterns rich in plant foods, fiber, and unsaturated lipids may influence pathways involved in brain aging and neurodegeneration [8,9]. They have also been associated with reduced oxidative stress and neuroinflammation, improved metabolic and vascular health, and better neurological outcomes in aging populations [10]. Moreover, dietary components may influence gut microbiota composition, gastrointestinal function, and nutrient bioavailability, factors that are increasingly implicated in PD pathophysiology and therapeutic response, including levodopa absorption [11]. However, these patterns are not only characterized by the presence of potentially protective food groups. They also restrict or negatively score food groups commonly associated with Western dietary patterns, including red and processed meats, sweets, fried foods, saturated fats, refined products, and ultra-processed foods [12,13]. Therefore, comparisons between the Amazonian Diet and these dietary models should consider both dimensions: the inclusion of bioactive-rich foods and the displacement of dietary components associated with higher inflammatory, metabolic, or oxidative burden.

Interestingly, several traditional dietary systems outside the Mediterranean region share comparable nutritional characteristics. In the Brazilian Amazon, particularly in riverine communities, traditional dietary practices still preserve elements of a Pre-Columbian dietary pattern commonly referred to as the Amazonian Diet. Although elements of the Western diet have gradually been incorporated, local food systems continue to rely heavily on a wide diversity of native fruits and freshwater fish, as well as cassava-derived products, maize, and other root crops. In addition, vegetables such as pumpkin and leafy greens are frequently consumed [14,15,16,17]. Epidemiological studies suggest that adherence to this dietary pattern may be associated with relevant metabolic outcomes. In a study involving 7041 adolescents, higher adherence to the Amazonian Diet was associated with a lower prevalence of high blood pressure, reduced insulin resistance, and a lower frequency of low HDL levels, although a higher prevalence of elevated triglycerides was also reported [18]. This dietary system is of particular interest because it combines ecological specificity, cultural continuity, and a high diversity of nutrient-dense and bioactive-rich foods within a minimally processed food matrix.

Many of these foods present in the Amazonian Diet are rich in micronutrients and bioactive compounds, including polyphenols, carotenoids, vitamins, and polyunsaturated fatty acids (PUFAs), which have been associated with antioxidant and anti-inflammatory properties relevant to neuroprotection [19,20,21,22]. Rather than acting on isolated molecular targets, these compounds may exert pleiotropic effects on interconnected pathways involved in neuronal maintenance and aging-related vulnerability [23].

Despite the biological richness of Amazonian foods, current evidence remains fragmented across individual foods, isolated compounds, and non-integrated mechanistic observations, with limited attempts to examine the Amazonian Diet as a biodiversity-based dietary pattern relevant to Parkinson’s disease.

The present study aimed to investigate the potential relevance of key foods derived from the Amazonian Diet to Parkinson’s disease by integrating compositional nutritional analysis, multivariate analytical approaches, and evidence synthesis focused on biological mechanisms related to neurodegeneration. Thus, this study proposes the Amazonian Diet as a biologically plausible, biodiversity-based dietary framework for exploring diet–neurodegeneration interactions in Parkinson’s disease.

However, this study does not assess whether the Amazonian Diet influences the incidence, progression, clinical symptoms, levodopa response, or disease-related biomarkers of Parkinson’s disease. It examines whether selected Amazonian Diet foods contain nutritional and bioactive constituents that intersect with biological pathways implicated in PD pathophysiology. Therefore, the Amazonian Diet is discussed here as a biologically plausible and hypothesis-generating dietary framework, not as a clinically validated intervention for Parkinson’s disease.

**Figure 1:**
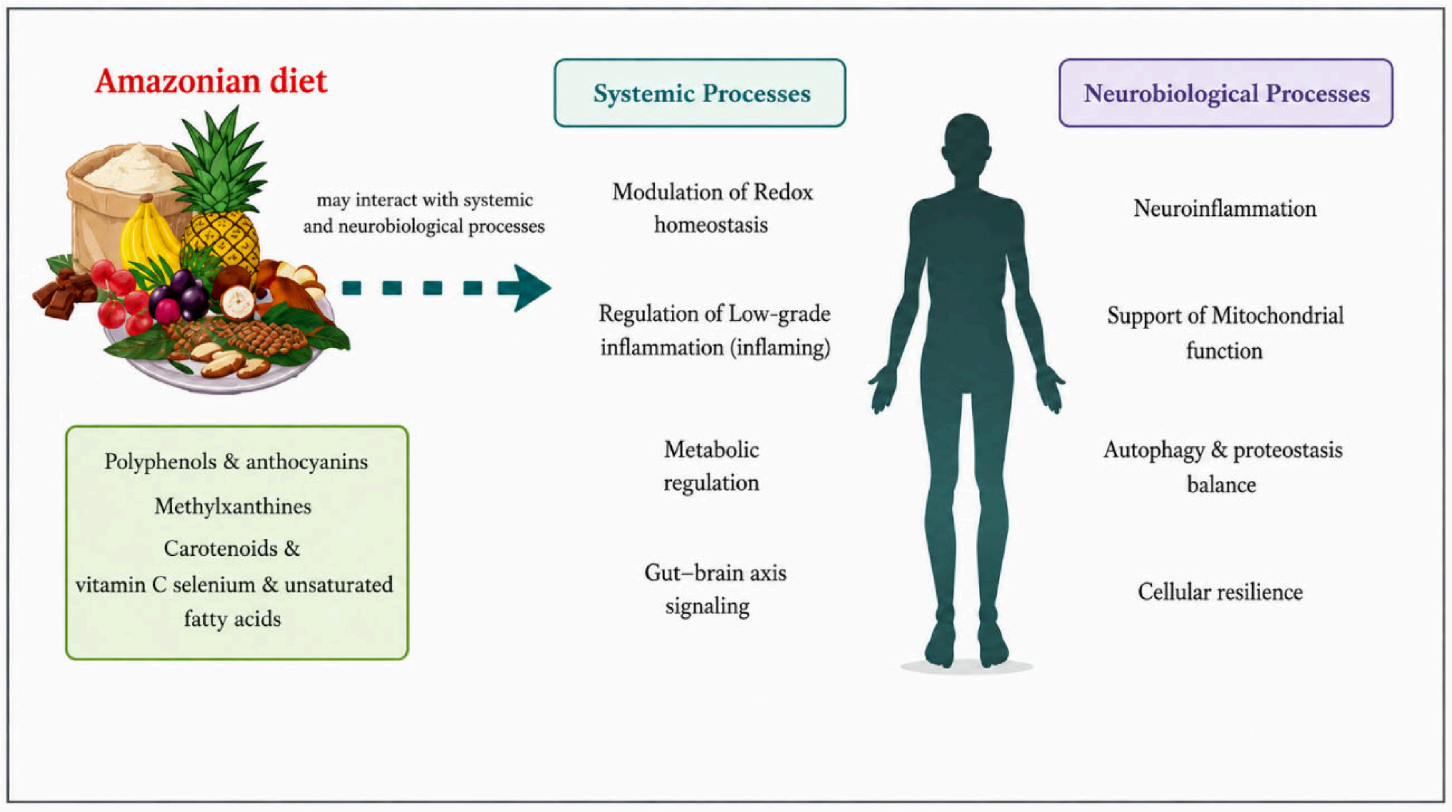
Conceptual framework linking Amazonian Diet components to biological pathways relevant to Parkinson’s disease. Traditional dietary patterns based on the Amazonian Diet include native fruits, seeds and nuts, freshwater fish, cassava-derived foods, and minimally processed regional foods. These foods provide a diverse nutritional matrix containing bioactive and nutritional compounds such as polyphenols, anthocyanins, flavonoids, carotenoids, unsaturated fatty acids, selenium, vitamins, and dietary fiber. Several of these compounds have been associated in experimental, compositional, or observational studies with biological processes also implicated in the pathophysiology of Parkinson’s disease, including oxidative stress, neuroinflammation, mitochondrial function, redox regulation, gut–brain axis signaling, and neuronal resilience.

To provide an integrated overview of the conceptual framework underlying this study, Figure 1 illustrates the relationship between key components of the Amazonian Diet, their bioactive compounds, and biological mechanisms involved in Parkinson’s disease.

## 2. Methodology

### 2.1. Study Design

The study was guided by the following research question: what is the potential role of key foods derived from the Amazonian Diet, and their associated bioactive compounds, in biological processes relevant to the pathophysiology of Parkinson’s disease and nutritional neuroscience?

For this, the study was designed as an exploratory study structured in two complementary stages comprising a compositional and multivariate analysis of bioactive compounds of foods derived from the Amazonian Diet and an integrative literature review of five selected fruits with mechanistic relevance to Parkinson’s disease (PD). It was not designed as a conventional systematic review or meta-analysis. First (Stage 1), a structured bibliographic mapping of Amazonian Diet foods was performed to identify studies addressing nutritional composition, chemical composition, bioactive compounds, and functional properties. This mapping supported the construction of the food composition and bioactive profile dataset used in the multivariate analysis. Second (Stage 2), an integrative mechanistic review was conducted to identify evidence directly or indirectly related to PD, neurodegeneration, and biological pathways implicated in PD pathophysiology. The two components were subsequently integrated to support a hypothesis-generating interpretation of the potential relevance of Amazonian Diet components to PD-related biological pathways.

This design was adopted because of the limited number of studies directly linking PD to Amazonian dietary patterns and the fragmented nature of the existing literature, which focuses predominantly on isolated compounds rather than integrated dietary models. Therefore, this hybrid strategy allowed the integration of nutritional analysis with evidence from bioactive compounds, functional properties, and neurodegeneration on experimental, observational, and conceptual studies.

#### 2.1.1. Stage 1—Food Selection and Nutritional Characterization of Amazonian Foods

To address this research question, a total of 36 foods were selected, comprising 24 plant-derived foods (22 fruits, cassava root, and Jambu leaves) and 12 fish species commonly consumed by Amazonian riverine populations [24,25]. The selection was supported by population-based studies among older riverine adults and by dietary surveys in Amazonian communities [13,15,16,24], ensuring that the analyzed items reflect actual regional dietary patterns. In an ethnonutritional context, one study of 492 individuals living in rural areas of the municipality of Coari (Amazonas, Brazil), intake was assessed using a brief Food Frequency Questionnaire and dietary patterns were identified by principal component analysis; the most prevalent patterns (“vegetables” and “traditional riverine”) encompassed the majority of the foods analyzed here, supporting the representativeness of the selected items [26].

All nutritional data are expressed per 100 g of edible portion, using values officially provided by the Brazilian Food Composition Table (TBCA) [27] and following Food and Agriculture Organization (FAO) guidelines [28], ensuring the reliability and standardization of the assessed data. Values corresponding to fresh foods (pulp or whole fruit) or cooked foods (particularly in the case of fish) were preferentially used.

For fruits, vegetables, and fish, data on energy (kcal), carbohydrates (g), proteins (g), lipids (g), and dietary fiber (g) were obtained considering their consumption either in natura (as pulp or juice) or after cooking. Protein, lipid, and fiber profiles of Amazonian foods were specifically analyzed. Particular attention was paid to proteins rich in large neutral amino acids (LNAAs) because they may interfere with the absorption and metabolism of levodopa, the gold-standard pharmacological treatment for PD [29,30]. Conversely, polyunsaturated fatty acids (PUFAs) and dietary fiber may exert beneficial effects through anti-inflammatory, metabolic, and gut–brain mechanisms relevant to PD [18,31].

Selected micronutrients and lipid fractions potentially relevant to PD pathophysiology were also compiled from TBCA and FAO, including zinc, magnesium, iron, copper, and selenium; vitamins A, B3, B6, B9 (folate), B12, C, and E; and saturated, monounsaturated (MUFAs), and polyunsaturated fatty acids (PUFAs) [32,33,34,35,36,37,38,39].

Exotic foods introduced and widely consumed in riverine communities, such as avocado [40], banana [41], coffee [42], mango [43,44], maize [45], pumpkin [46,47], and leafy green kale [48], were not included in the analysis due to the very large number of studies available on these items. Nevertheless, these foods are also considered in the discussion of the results, based on previously published literature reviews.

A dedicated bioactive compound literature review was not performed for Amazonian fish species: their components of principal interest for PD—fatty acids and proteins—were characterized through compositional (proximate) data. This decision is acknowledged as a limitation, and fish-derived omega-3 considerations are therefore treated as compositional/background evidence and are not assigned the same evidentiary status as the reviewed plant-derived bioactives. To our knowledge, no included study directly evaluated Amazonian fish intake in Parkinson’s disease models or clinical PD outcomes.

#### 2.1.2. Stage 1—Characterization of Bioactive Molecules Amazonian Diet Foods (Search 1)

**Figure 2:**
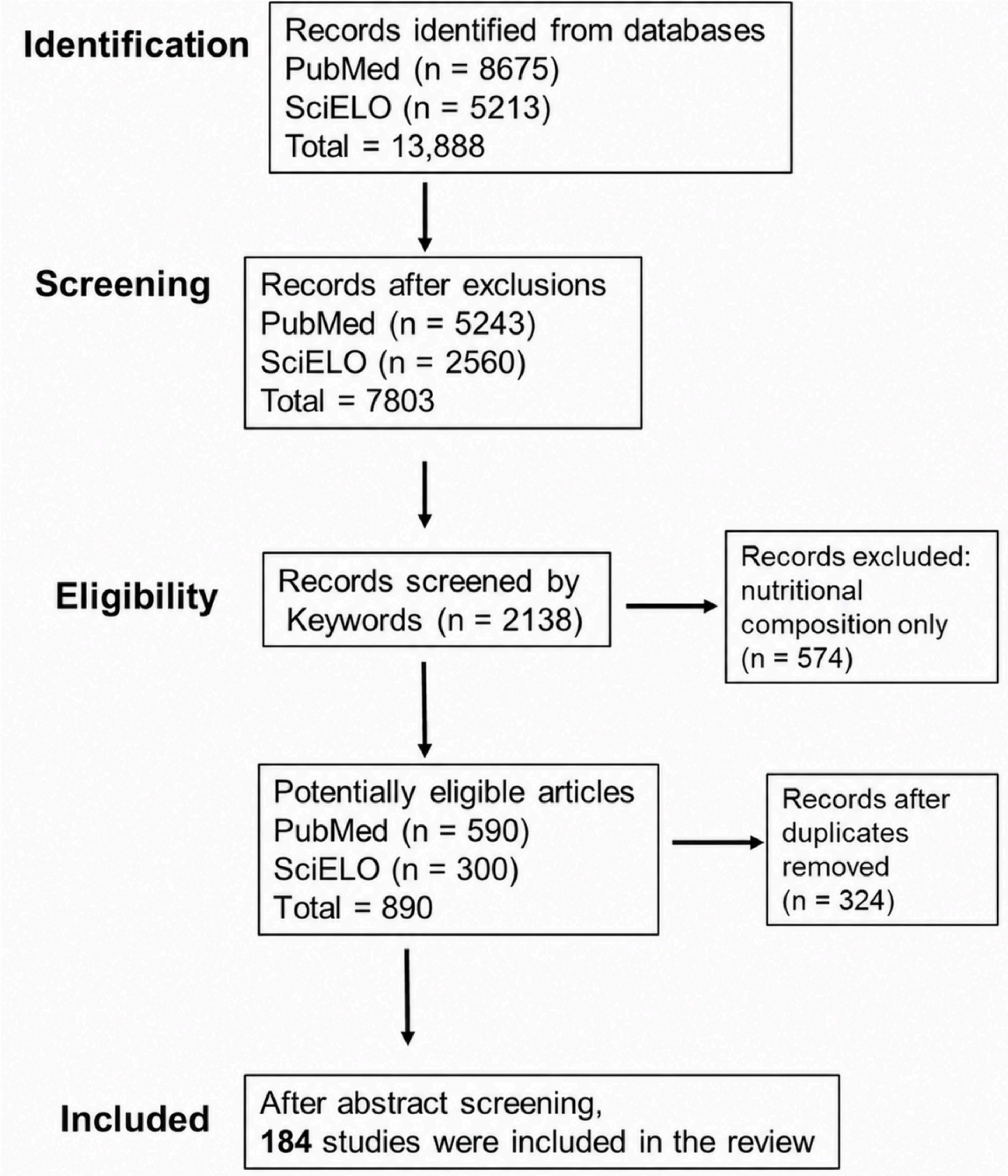
PRISMA-informed flow diagram of the literature search and study selection process. Flowchart illustrates the identification, screening, eligibility assessment, and inclusion of studies retrieved from the PubMed and SciELO databases. Records were filtered according to predefined exclusion criteria (e.g., non-English language, review articles, studies on by-products, cosmetic or nanoformulated products, and plant breeding), followed by screening using keywords related to nutritional composition, bioactive compounds, functional properties, and Parkinson’s disease (PD). The final set of publications included in the integrative analysis (Stage 1) is shown.

The analysis of bioactive compounds and functional properties of fruits and other plant-based foods potentially relevant to PD was conducted through a structured study selection process. Identification, screening, eligibility, and inclusion stages were summarized using a PRISMA-informed evidence-mapping approach (Figure 2) adapted from the PRISMA 2020 guidelines [49].

This search was conducted in two databases, PubMed/MEDLINE and SciELO, focused on studies involving Amazonian plant-derived foods that investigated bioactive molecules and biological mechanisms potentially relevant to PD, such as polyphenols, carotenoids, and miscellaneous compounds found only in certain species. In addition to these components, studies reporting biological properties with putative relevance to PD-related mechanisms were also considered, including antioxidant, anti-inflammatory, metabolic bioenergetic modulation, neuroprotective (including anti-apoptotic), and neurofunctional activities. Additionally, evidence from experimental models of PD including exposure to MPTP (1-methyl-4-phenyl-1,2,3,6-tetrahydropyridine), 6-hydroxydopamine (6-OHDA), rotenone, or paraquat [50,51] were analyzed and grouped as “PD-related mechanistic activity”.

Studies were excluded if they were (i) reviews, meta-analyses, or purely in silico; (ii) not written in English; (iii) duplicates or clearly unrelated to the selected foods in the title or abstract; or (iv) concerned with residues, by-products, non-edible parts, supplements, cosmetics, or nano-structured/encapsulated compounds.

Data extraction was performed using a predefined extraction form including food/compound, study design, model, dose or exposure when available, biological outcome, PD-specific or non-specific mechanism, and evidence category. Extracted data were checked by a second reviewer, and disagreements were resolved by consensus.

Considering that there is no single standardized method for the extraction and quantification of chemical compounds, studies related to proximate composition were excluded. Only macro- and micronutrient data obtained from TBCA and FAO were used. Due to the variable range of analytical methods across studies, quantitative values were not extracted; instead, a qualitative approach (presence-based) was adopted to ensure comparability of bioactive profiles.

The PRISMA-informed flow diagram presented in this manuscript refers specifically to Stage 1, which was designed as a broad evidence-mapping step aimed at identifying studies on Amazonian foods, including nutritional composition, chemical composition, bioactive compounds, and functional properties. Stage 2 did not represent a second independent PRISMA-based systematic review process; rather, it consisted of a focused integrative synthesis of mechanistic evidence related to the five foods prioritized after Stage 1.

#### 2.1.3. Stage 1—Data Analysis

All statistical analyses and visualizations were performed in R version R 4.3.2 (R Foundation for Statistical Computing, Vienna, Austria) [52]; data organization used Microsoft Excel. This integrative approach enabled the identification of potential relationships between Amazonian foods, their bioactive compounds, and biological mechanisms associated with neurodegenerative processes. The absence of formal sensitivity analyses using alternative distance metrics or variable combinations is acknowledged as a limitation.

In the Amazonian fruits analyzed in this study, bioactive molecules and biological properties were standardized using z-score transformation to eliminate scale differences and allow direct comparison across variables with different measurements.

Because absence of reporting does not necessarily indicate true absence, unreported bioactive compounds were coded as “not documented” rather than biologically absent. For exploratory visualization, undocumented compounds were treated as zero only within the presence-based richness matrix, and this assumption was acknowledged as a limitation.

Hierarchical clustering analysis was performed using Euclidean distance as the dissimilarity metric and Ward’s linkage method, which minimizes within-cluster variance and is widely used in nutritional and multivariate datasets. The clustering matrix included the following variables: dietary fiber, protein content, vitamin richness score, mineral richness score, polyphenol richness score, carotenoid richness score, and miscellaneous bioactive richness score. Macronutrients were entered as continuous variables, whereas bioactive classes were coded as presence-based richness scores.

Cluster structure was visualized using a dendrogram with color-coded branches. To provide a complementary representation of similarity patterns, a combined dendrogram–heatmap (clustermap) was generated, allowing the simultaneous visualization of hierarchical relationships among foods and the relative contribution of each variable. This approach aimed to identify integrated nutritional signatures and similarity patterns among foods, supporting the interpretation of dietary structures rather than isolated components. These exploratory analyses were conducted to identify integrated nutritional signatures and not to establish causal relationships.

#### 2.1.4. Stage 2—Selection of Amazonian Fruits and Seeds as Sources of Bioactive Compounds for the Integrative Literature Review

From the foods characterized in Stage 1, five fruits and seeds—açaí (*Euterpe oleracea*), cocoa/cacao (*Theobroma cacao*), camu-camu (*Myrciaria dubia*), guaraná (*Paullinia cupana*), and Brazil nuts (*Bertholletia excelsa*)—were selected for mechanistic interpretation in Stage 2 based on three converging criteria: (i) their positioning in the exploratory hierarchical clustering, where they appeared among foods with greater documented bioactive diversity and relevant nutritional attributes; (ii) the comparatively greater availability of experimental, preclinical, observational, or mechanistic evidence related to neurodegeneration or PD-relevant biological pathways; and (iii) their cultural and ethnonutritional relevance within Amazonian dietary patterns.

Cultural and ethnonutritional relevance was defined based on documented use in Amazonian dietary surveys, traditional consumption reports, and representation within riverine dietary patterns, rather than on global commercial popularity [12,14,16,24]. Therefore, the selection of these five foods should be understood as an evidence-informed and hypothesis-generating prioritization for mechanistic discussion, rather than evidence that these foods have demonstrated clinical efficacy or disease-modifying effects in Parkinson’s disease.

#### 2.1.5. Stage 2—Integrative Review of Mechanistic Evidence of Amazonian Fruits and Seeds (Search 2)

A second, complementary bibliographic mapping process was conducted to identify studies directly or indirectly related to PD, neurodegeneration, and biological mechanisms implicated in PD pathophysiology. This stage focused particularly on the Amazonian fruits and seeds as described above.

The search strategy was developed sequentially. First, a direct search combining terms related to the Amazonian Diet and Parkinson’s disease was performed. This initial search did not identify studies explicitly evaluating the Amazonian Diet as a dietary pattern in relation to PD. A broader exploratory search combining terms related to Parkinson’s disease, neurodegeneration, nutrition, dietary patterns, antioxidant activity, anti-inflammatory activity, mitochondrial dysfunction, and neuroprotection retrieved a highly heterogeneous body of literature. Therefore, the search strategy was refined using conceptual blocks combining (i) Parkinson’s disease, pathophysiology, and nutritional aspects; (ii) dietary patterns, aging, and neuroprotection; (iii) Amazonian foods, chemical composition, and bioactive compounds; and (iv) the specific selected foods and their botanical names.

Studies were considered eligible for mechanistic interpretation if they addressed at least one of the following aspects: experimental or observational evidence related to oxidative stress, neuroinflammation, mitochondrial function, dopaminergic signaling, autophagy, apoptosis, metabolic regulation, or other pathways implicated in PD pathophysiology; neurological, neurodegenerative, psychiatric, chronic inflammatory, metabolic, or cardiovascular outcomes relevant to the interpretation of PD-related mechanisms; or direct PD experimental models, including MPTP, 6-OHDA, rotenone, or paraquat models.

Eligible studies were categorized according to the proximity of their evidence to PD. Four evidence categories were defined: (1) studies directly related to PD, including experimental PD models, PD-related mechanisms, or human evidence involving PD; (2) studies indirectly related to PD through evidence involving other neurological, neurodegenerative, or psychiatric conditions, such as Alzheimer’s disease, cognitive decline, depression, or other brain-related outcomes; (3) studies related to other chronic or systemic conditions, including cardiovascular, metabolic, inflammatory, or oxidative stress-related diseases; and (4) studies reporting nutritional composition, chemical composition, or functional properties without disease-specific outcomes.

Data extraction was performed using a predefined form including the selected food or compound, study design, biological model, dose or exposure when available, disease or condition investigated, main reported outcome, PD-specific or non-specific mechanism, and evidence category. Extracted data were checked by a second reviewer, and disagreements were resolved by consensus.

Contextual references on Mediterranean, DASH, and MIND dietary patterns; general PD pathophysiology; levodopa–protein interactions; omega-3 fatty acids; gut–brain axis mechanisms; and nutritional neuroscience were incorporated to support the conceptual framework and interpretation of findings. These contextual references were not treated as part of the primary compositional mapping unless they also met the predefined eligibility criteria.

### 2.2. Integration of Compositional and Mechanistic Evidence

The final interpretation was based on the integration of the two bibliographic mapping processes with the multivariate nutritional analysis. From the foods characterized in the compositional dataset, five fruits and seeds—açaí, cocoa/cacao, camu-camu, guaraná, and Brazil nuts—were prioritized based on three converging criteria: (i) their position in the exploratory hierarchical clustering as foods with high bioactive diversity and relevant nutritional attributes; (ii) the availability of experimental, preclinical, observational, or mechanistic evidence linking their constituents to PD-related or neurodegeneration-related pathways; and (iii) their cultural and ethnonutritional relevance within Amazonian dietary patterns.

This integrated approach allowed the manuscript to distinguish compositional evidence from mechanistic evidence and to separate direct PD-related evidence from indirect evidence derived from other neurodegenerative, chronic, or systemic disease models. The resulting interpretation is therefore hypothesis-generating and should not be understood as evidence of clinical efficacy, disease prevention, or disease modification in Parkinson’s disease.

### 2.3. Evidence Grading and Synthesis

Given the intentional integration of heterogeneous evidence, each source was labeled by evidence type, either direct or indirect mechanistic evidence, in relation to PD. This classification is reported alongside the synthesized findings and presented in Supplementary Material Table S5, so that compositional and mechanistic evidence can be clearly distinguished from observational and clinical evidence.

Because this review was designed as an exploratory and hypothesis-generating synthesis rather than as a systematic review aimed at estimating intervention effects, a formal risk-of-bias assessment was not performed. Instead, evidence was interpreted according to study type, biological proximity to PD, and translational strength. Consequently, compositional data, in vitro findings, animal toxin model evidence, observational human studies, and clinical trials were not treated as equivalent levels of evidence.

## 3. Results and Discussion

### 3.1. Nutrients from Foods Derived from Amazonian Diet with Relevnace to Parkinson’s Disease

The macronutrient composition of foods derived from the Amazonian Diet demonstrates a heterogeneous nutritional profile, as summarized in Table 1 representing foods in their commonly consumed edible forms.

Amazonian Diet foods are generally characterized by high water content and relatively low energy density (typically ranging from 18 to 90 kcal per 100 g), with moderate amounts of carbohydrates and dietary fiber. Notable exceptions include energy-dense fruits such as Brazil nuts (674 kcal/100 g), buriti (207 kcal/100 g), peach palm (212 kcal/100 g), and tucumã (262 kcal/100 g), which present substantially higher lipid content.

In contrast, freshwater fish species represent the primary sources of dietary protein and lipids within this dietary pattern, with protein values typically ranging from approximately 19 to 25 g per 100 g of edible portion and negligible carbohydrate content.

Overall, the nutritional profiles of these foods reflect the diversity of plant and animal species that compose the Amazonian Diet, combining fruits with relatively low energy density and staple foods that provide carbohydrates and protein. This pattern may contribute to a more balanced distribution of macronutrients and could influence metabolic and neurological processes associated with aging and neurodegenerative diseases, including PD. Indeed, evidence suggests that dietary composition can modulate key mechanisms involved in PD pathophysiology, such as oxidative stress and neuroinflammation [11,17,18,21,53].

### 3.2. Proteins

Among macronutrients, dietary protein plays a particularly relevant role in the context of PD due to its interaction with levodopa therapy. Levodopa, the main pharmacological treatment for PD, shares intestinal and blood–brain barrier transport systems with large neutral amino acids (LNAAs), including leucine, isoleucine, valine, phenylalanine, and tyrosine. As a result, higher dietary protein intake may compete with levodopa’s absorption and transport, potentially reducing its bioavailability and affecting motor symptom control [27,28].

Within this context, the interaction between LNAAs and levodopa has important clinical implications, particularly in the context of dietary patterns such as the Amazonian Diet, in which fish constitutes a major protein source. The distribution and timing of protein intake may therefore represent a relevant nutritional strategy for optimizing therapeutic response in PD, as supported by a previous study [27]. Table 2 summarizes the approximate protein content of major food groups commonly consumed in the Amazonian Diet and provides general considerations regarding their compatibility with daytime levodopa administration.

### 3.3. Lipids

Beyond protein-related interactions, dietary lipids, particularly polyunsaturated fatty acids (PUFAs), are critically involved in neuronal structure and function and have been associated with the modulation of neurodegenerative processes. Epidemiological evidence, including large-scale population studies such as those conducted by Gu et al. [19], indicates that lower dietary intake of *n*-6 PUFAs is associated with an increased risk of neurodegenerative diseases, including PD. These findings support the role of dietary lipid composition as a relevant factor in neurological health.

In the present analysis of the PUFA profile of Amazonian fish, several Amazonian freshwater fish species, including tucunaré, pirarucu, Amazonian croaker, and small pelagic “Amazonian sardines,” exhibited favorable lipid profiles characterized by higher levels of long-chain omega-3 fatty acids (EPA + DHA) and balanced omega-3/omega-6 ratios, as shown in Table 3. These characteristics are associated with anti-inflammatory effects, stabilization of neuronal membranes, and modulation of neurodegenerative pathways. Although marine fish are traditionally considered the main sources of long-chain omega-3 fatty acids, these findings indicate that Amazonian freshwater species may also provide relevant contributions to dietary lipid intake.

Comparative studies indicate that species such as tambaqui, pirarucu, and matrinxã may provide EPA and DHA levels comparable to those for commonly consumed freshwater fish from other regions, including carp and tilapia, and in some cases approaching those observed in marine species such as sardine and mackerel [54]. Moreover, the omega-6/omega-3 ratios reported in several Amazonian species fall within ranges considered nutritionally favorable, which have been associated with reduced inflammatory responses, improved oxidative balance, and potential benefits for neurological health [19].

These findings suggest that Amazonian freshwater fish may represent an important regional source of long-chain omega-3 fatty acids, contributing to dietary lipid patterns that could influence mechanisms implicated in neurodegenerative diseases.

In addition to this, the lipid profile of foods derived from the Amazonian Diet—characterized by the combined presence of monounsaturated fatty acids from plant sources and long-chain omega-3 fatty acids from fish—supports a dietary pattern with the potential to modulate inflammatory processes, preserve neuronal membrane integrity, and potentially influence the mechanisms involved in PD pathophysiology [19,52].

**Table 3:** Omega-3 and omega-6 fatty acid profile of Amazonian fishes and neuro-nutritional implications for Parkinson’s disease (PD).

Amazonian Fishes	Omega-3 (EPA + DHA)	Omega-6	Omega-3/Omega-6 Ratio	Potential Relevance for PD	Neuro-Nutritional Remarks
Amazonian sardine (*Triportheus*)	H	MOD	Favorable	H	Good DHA source; whole consumption provides minerals
Armored catfish (*Loricariidae*)	L	MOD	Unfavorable	L	Low total lipid content and low omega-3
Curimatã (*Prochilodus* spp.)	L	H	Unfavorable	L	Predominantly linoleic acid
Jaraqui (*Semaprochilodus* spp.)	L	H	Unfavorable	L	Plant-based dietary profile
Mapará (*Hypophthalmus edentatus*)	MOD	MOD	Intermediate	MOD	Fatty fish with high energy value
Matrinxã (*Brycon amazonicus*)	MOD	H	Intermediate	MOD	Fruit-based diet increases omega-6
Pirapitinga (*Piaractus brachypomus*)	MOD	H	Unfavorable	L-MOD	Lipids derived from seeds
Pacu (*Piaractus mesopotamicus*)	MOD	H	Unfavorable	L-MOD	Similar to tambaqui
*Amazonian croaker*	H	L	Favorable	H	Profile similar to marine omega-3-rich fish
Pirarucu (*Arapaima gigas*)	H	MOD	Favorable	VH	Rich in DHA; high-quality protein
Tambaqui (*Colossoma macropomum*)	MOD	VH	Unfavorable	L-MOD	Very energy-dense; omega-6 predominant
Tucunaré (*Cichla* spp.)	H	L	Very favorable	VH	Excellent source of DHA and EPA

VL = very low; L = low; MOD = moderate; H = high; VH = very high; main references: [52,55,56,57,58,59].

### 3.4. Fibers

Dietary fiber represents another key nutritional component with potential relevance to PD, particularly regarding the gut–brain axis. Several Amazonian plant-derived foods simultaneously provide fiber, micronutrients, and bioactive compounds. These foods form a complex nutritional matrix rather than isolated nutrient sources, as presented in Table 1. This characteristic is consistent with current perspectives emphasizing dietary patterns, rather than individual nutrients, as modulators of neurodegenerative processes. Higher dietary fiber intake has been associated with increased abundance of butyrate-producing bacteria and improved intestinal barrier function, both of which support the idea intestinal epithelial integrity is relevant to the modulation of systemic and neuroinflammatory responses in PD and its progression [60,61].

From a mechanistic perspective, plant-based dietary patterns rich in fiber and bioactive molecules have been suggested to exert neuroprotective effects, partly through microbiota-mediated mechanisms [17]. Gastrointestinal dysfunction, including constipation and microbial dysbiosis, is among the most common non-motor features of PD and may precede motor symptoms. Within this context, dietary fiber plays a central role as a fermentable substrate for gut microbiota, promoting the production of short-chain fatty acids. These metabolites contribute to the maintenance of intestinal integrity, modulation of immune responses, and regulation of signaling pathways within the microbiota–gut–brain axis [59].

### 3.5. Bioactive Molecules and Potential Biological Properties of Amazonian Fruits/Vegetables with Potential Beneficial Effect on Parkinson Disease Modulation

The bioactive profile of Amazonian Diet foods, detailed in Table 4, shows a rich and multifaceted molecule composition with potential intersection with the biological pathways implicated in PD pathophysiology. These foods provide a wide spectrum of bioactive compounds, including polyphenols, carotenoids, vitamins, essential minerals, and bioactive lipids, which have been implicated in the modulation of oxidative stress, neuroinflammation, mitochondrial dysfunction, and neuronal survival. The convergence of these compounds within a single dietary pattern supports the hypothesis that the Amazonian Diet may exert synergistic effects on biological pathways associated with neurodegeneration.

The only fruit showing a potentially adverse effect in PD was soursop (*Annona muricata*), due to the presence of acetogenins in its chemical matrix, which are potent inhibitors of mitochondrial Complex I [62]. For this reason, soursop was excluded from subsequent multifactorial and integrative analyses.

**Figure 3:**
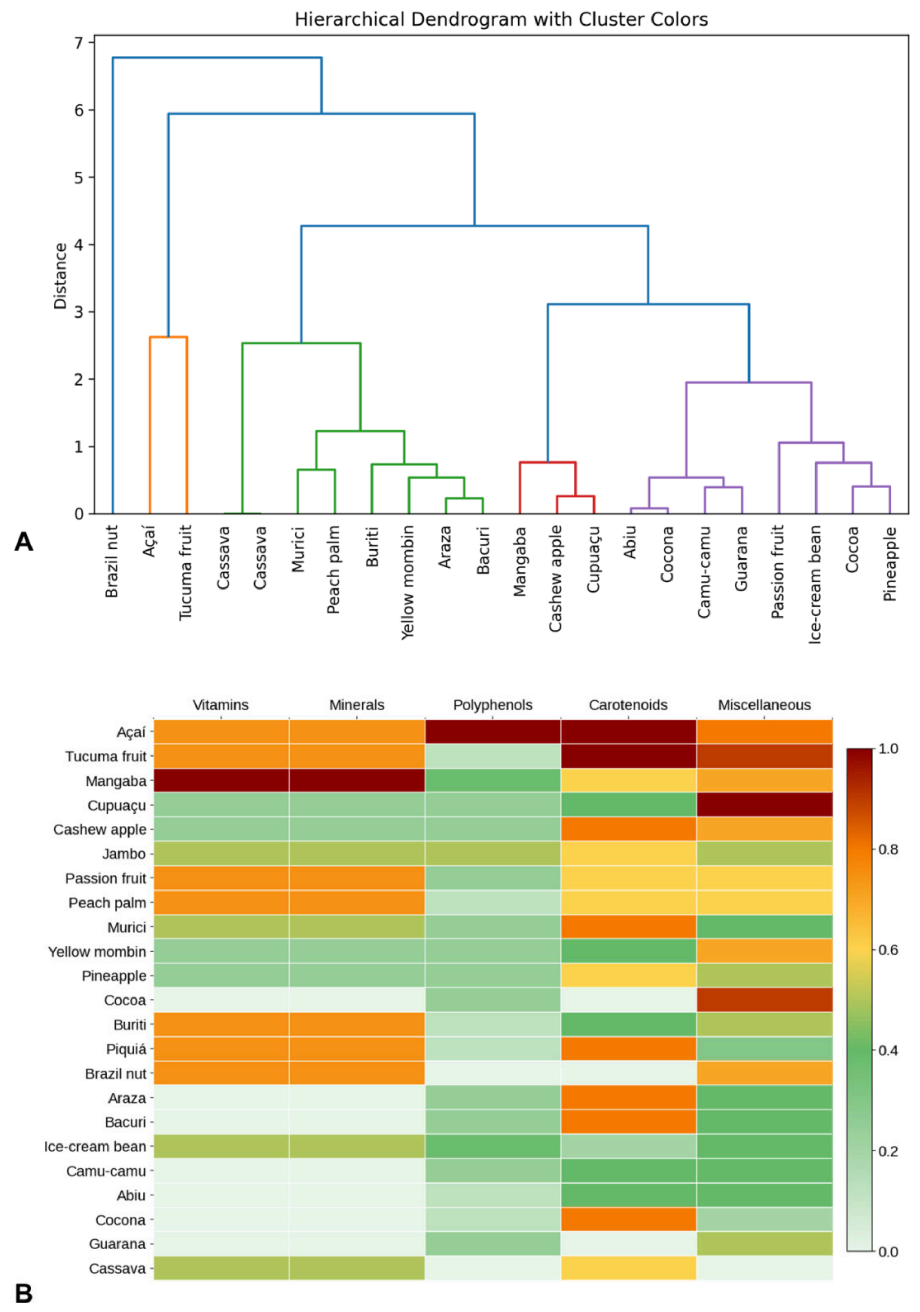
Multivariate characterization of nutritional and bioactive profiles of Amazonian plant foods. (**A**) Hierarchical clustering dendrogram showing similar patterns based on fiber content, protein levels, and overall bioactive richness. (**B**) Heatmap displaying the relative distribution of major bioactive compound classes across foods. Color intensity indicates relative abundance. The combined analyses highlight marked heterogeneity among foods and identify clusters with potentially greater relevance to Parkinson’s disease-related mechanisms.

The multivariate analysis revealed marked heterogeneity in the nutritional and bioactive profiles of Amazonian Diet-derived foods. Hierarchical clustering based on dietary fiber, protein content, and bioactive compound diversity identified distinct groups of foods with integrated compositional patterns (Figure 3A). A clearly defined cluster characterized by high values across multiple dimensions was distinctly separated from the others, suggesting a dense nutritional and phytochemical profile with possible interaction with pathways implicated in neurodegenerative disorders. This group included lipid-rich palm fruits and nuts, particularly acai berry, buriti fruit, tucumã fruit, peach palm, and Brazil nuts, which are known to concentrate energy-dense nutrients alongside a wide diversity of bioactive compounds.

**Table 4:** Main bioactive molecules of Amazonian fruits/vegetables with potential beneficial effect on Parkinson’s disease.

Foods	Polyphenols			Miscellaneous				Ref
	Vitamins/ Minerals *	Major Flavonoids	Others	Major Carotenoids	Type	Main Molecules	Main Bioactive Properties	
Abiu	C	Quercetin	Gallic Ac, Ellagic acid, Chlorogenic Ac, Caffeic Ac.	β-carotene, lutein			Antioxidant, anti-inflammatory	[63,64,65,66]
*Acai berry*	C, E, Fe, Mg	Cyanidin-3-, catechin, epicatechin, orientin, isovitexin, apigenin, Chrysin, luteolin	Gallic Ac, Protocatechuic Ac, Ferulic Ac, p-Hydroxybenzoic Ac.	Lutein, zeaxanthin, α-carotene, β-carotene	FA	Ω-9, Ω-6, Ω-3	Antioxidant, anti-inflammatory, mitochondrial modulation neuroprotective, PD modulation	[67,68,69,70,71,72,73,74,75,76,77,78,79,80,81,82,83,84,85,86,87,88,89,90]
*Araza*	C	Quercetin, myricetin	Gallic Ac, Protocatechuic Ac, Chlorogenic Ac, Caffeic Ac,)	Lutein, β-cryptoxanthin, a, zeinoxanthin			Antioxidant, antimicrobial, antinoceptive	[91,92,93,94,95]
*Bacuri fruit*	C	Catechin, quercetin	Gallic Ac, Chlorogenic Ac, Caffeic Ac, Ferulic Ac.	α -carotene, β-carotene, lutein, zeaxantin	.		Antioxidant, anti-inflammatory, antimicrobial	[96,97,98,99,100]
*Brazil nuts*	E, Mg, Zn, Cu, Se	Minor flavonoids	Gallic Ac, p Protocatechuic Ac, 2 Hydroxybenzoic Ac, *p*-coumaric Ac, Sinapic Ac.		FA	Ω-6, Ω-9	Antioxidant, Neuroprotection, Bioenergetic, PD modulation	[101,102,103,104]
*Buriti fruit*	A, C, E, Mg	Quercetin	Gallic Ac, Protocatechuic Ac, Caffeic Ac.	β-carotene (very high), lutein	FA	Ω-9, Ω-6	Antioxidant, immune support	[105,106,107,108,109,110,111]
*Cocoa*	C	Catechin, epicatechin	Gallic Ac, Ellagic acid, Caffeic Ac, Ferulic Ac, Anacardic Ac.		Alkaloids	Caffeine, theobromine, Ω-9, Ω-6	Antioxidant, neuroprotective, anti-inflammatory, PD modulation	[112,113,114,115,116,117]
*Cashew apple*	A, C	Quercetin, kaempferol	Gallic Ac, Ellagic acid, Chlorogenic Ac, Caffeic Ac, Proanthocyanidins	β-carotene, lutein, violaxanthin, zeaxanthin	Phenolic lipids	Ω-9, Ω-6	Antioxidant, anti-inflammatory, antimicrobial, neuroprotective, PD modulation	[118,119,120,121,122,123,124,125,126,127,128]
*Camu-camu*	C	Quercetin, myricetin	Chlorogenic Ac, Caffeic Ac, Gallic Ac, Caffeoylquinic Ac.	β-carotene (trace), lutein (trace)			Antioxidant, anti-inflammatory, immunomodulatory, analgesic.	[129,130,131,132,133,134,135,136,137]
*Cocona*	B6	Quercetin		β-carotene, lutein, zeaxanthin, lycopene	FA	Ω-6, Ω-9	Antioxidant, metabolic regulation, hypoglycemic	[138,139,140]
Cupuaçu	B6, C	Catechin, epicatechin	Gallic Ac, Ellagic acid, Caffeic Ac, heograndins (unique polyphenols), Isoscutellarein, Hypolaetin, Clovamide	β-carotene	Alkaloids	Ω-9, Ω-6, theobromine	Antioxidant, anti-inflammatory,	[141,142]
*Guarana*	A	Catechin, epicatechin	Gallic Ac, Ellagic Ac, Tannins		Alkaloids	Caffeine, theobromine theophylline	Antioxidant, anti-inflammatory, neuroprotective, bioenergetic, PD modulation	[143,144,145,146,147]
Ice-cream bean	B6, B9, C	Quercetin	Gallic Ac, Chlorogenic Ac, Caffeic Ac, Anthocyanins	β-carotene			Antioxidant, anti-inflammatory	[148,149]
*Mangaba fruit*	A, B9, C, E, Mg	Quercetin, rutin, catechin	Gallic Ac, Chlorogenic Ac, Caffeic Ac, Ellagic acid, Proanthocyanidins, Procyanidins *C*-glycosides	β-carotene, lutein, zeaxanthin			Antioxidant, anti-inflammatory,	[150,151,152,153]
*Murici fruit*	A, C, E	Quercetin, catechin	Gallic Ac.	β-carotene, lutein, zeaxanthin, β-cryptoxanthin	FA	Ω-6, Ω-3, Ω-9	Antioxidant, anti-inflammatory	[78,154,155,156]
*Passion fruit*	A, C, E, Mg	Isovitexin, vitexin	Gallic Ac, Chlorogenic Ac, Caffeic Ac, Ferulic Ac.	β-carotene, lutein, zeaxanthin	FA	Ω-9, Ω-6	Antioxidant, anti-inflammatory, mild sedative, PD modulation	[157,158,159,160]
*Peach palm*	A, B6, C, E	Quercetin	Gallic Ac, Chlorogenic Ac, Caffeic Ac.	β-carotene, α-carotene, lutein,	FA	Ω-9, Ω-6	Antioxidant, bioenergetic	[161,162]
*Pineapple*	C, B9	Quercetin, catechin	Gallic Ac, Chlorogenic Ac, Caffeic Ac, Ferulic Ac.	β-carotene, lutein, ll-E)-violaxanthin,	Proteolytic enzymes	Bromelain	Antioxidant, anti-inflammatory, PD modulation	[163,164,165,166,167]
*Piquia fruit*	A, B9, C, E	Quercetin	Gallic Ac, Ferulic Ac, *p*-Coumaric Ac.	β-carotene, α-carotene, lutein, zeaxanthin			Antioxidant, genoprotective	[168,169,170]
*Soursop*	B6, C	Quercetin, kaempferol	Gallic Ac, Chlorogenic Ac, Caffeic Ac.	β-carotene (trace), lutein (trace)	Acetogenins	Annonacin, bullatacin, annomuricin, annonacinone	Acetogenins are potent inhibitors of mitochondrial Complex I. Negative effect on PD	[62,171]
*Yellow mombin*	B9, C	Quercetin, kaempferol	Gallic Ac, Chlorogenic Ac, Caffeic Ac, Ferulic Ac, Isoferulic acid, *p*-coumaric Ac.	β-carotene, lutein	FA	Ω-9, Ω-6	Antioxidant, anti-inflammatory, antimicrobial	[172,173,174,175]
*Tucuma fruit*	A, B6, C, E	Quercetin	Gallic Ac, Chlorogenic Ac, Caffeic Ac, Ferulic Ac, Ellagic acid, Tannins, *p*-Coumaric.	β-carotene, α-carotene, lutein, zeaxanthin, β-cryptoxanthin	Cyanogenic glycosides	Linamarin, lotaustralin	Antioxidant, neuroprotective, anti-inflammatory, antimicrobial	[176,177,178,179,180,181]
*Cassava*	B6, B9, C			β-carotene, lutein, zeaxanthin			Carbohydrate-rich staple food; bioenergetic	
*Jambu leaf*	B6, B9, C	Quercetin, kaempferol, luteolin, apigen	Chlorogenic Ac, Caffeic Ac, Ferulic Ac, *p*-coumaric Ac.	β-carotene, lutein, zeaxanthin	*N*-Alkylamides	Spilanthol	Antioxidant, neuroprotective, anesthetic, sialagogue	[182,183,184,185]

* Relevant micronutrients were considered ≥5% RDA per 100 g edible portion, * Micronutrients providing ≥ 5% of the recommended dietary allowance (RDA) per 100 g edible portion. Vitamins are indicated by letter (A, B6, B9, C, E).

A second cluster comprised fruits with a high diversity of bioactive compounds but relatively low protein content, including mangaba fruit, murici fruit, bacuri fruit, araza, cupuaçu, and cashew apple, indicating a profile predominantly driven by antioxidant and anti-inflammatory properties. An intermediate cluster displayed moderate levels across all variables, suggesting a more balanced but less pronounced functional profile; this group included camu-camu, cocona, guarana, ice-cream bean, and passion fruit. In contrast, a separate cluster characterized by low protein content and comparatively reduced bioactive diversity, including abiu fruit, pineapple, cassava, and jambu leaf (rose apple), may exert more limited or more specific contributions to biological processes related to PD.

The cluster map corroborated the hierarchical structure and illustrated the relative contribution of each variable to cluster formation (Figure 3B). Foods located in the upper portion of the heatmap exhibited simultaneously high bioactive compound diversity and moderate-to-high fiber content, whereas those in lower positions showed reduced values across one or more variables. Overall, these findings suggest that nutritional relevance in the context of Parkinson’s disease is unlikely to be driven by a single dietary component, but rather by the integrated and potentially synergistic contribution of multiple nutrients and bioactive compounds.

Experimental evidence from in vitro and in vivo models suggests that a subset of eight Amazonian plant-derived foods may exert modulatory effects on PD. These include acai berry, guaraná, cocoa, Brazil nuts, cashew apple, passion fruit, and pineapple. Although available studies on pineapple are largely limited to the activity of bromelain [161,164]. Across all analytical approaches, a consistent pattern emerged of Amazonian foods having a multifactorial nutritional profile characterized by substantial dietary fiber, meaningful protein contribution, and a high diversity of bioactive compounds. These features are aligned with biological mechanisms implicated in PD, including modulation of the gut–brain axis, oxidative stress, neuroinflammation, and dopaminergic function [186]. The results therefore suggest that the potential neuroprotective value of traditional Amazonian diets may arise from the combined effects of multiple nutrients and bioactive compounds rather than from isolated dietary components.

Thus, the available evidence sets the foods derived from the Amazonian Diet, particularly fruits and seeds, as biologically plausible contributors within dietary patterns capable of interacting with mechanisms involved in PD pathophysiology. While current evidence does not support direct clinical recommendations for PD prevention or treatment, the compositional characteristics of these foods, combined with traditional dietary practices and experimental findings, provide a coherent rationale for their inclusion in integrative and mechanistically oriented research on nutrition, aging, and neurodegeneration within the conceptual framework of the Amazonian Diet.

Based on these clustering patterns, selected fruits with distinct and complementary functional profiles were prioritized for further mechanistic investigation in Stage 2.

### 3.6. Selected Amazonian Diet-Derived Fruits and Seeds as Sources of Bioactive Compounds with Mechanistic Relevance to Parkinson’s Disease

Among the foods derived from the Amazonian Diet, five selected fruits and seeds, including acai berry (*Euterpe* spp.), cocoa (*Theobroma cacao*), camu-camu (*Myrciaria dubia*), guaraná (*Paullinia cupana*), and Brazil nuts (*Bertholletia excelsa*), were prioritized, as described before. These foods consistently clustered as having a high diversity of bioactive compounds combined with relevant nutritional attributes, supporting their selection for further mechanistic investigation in Stage 2. These fruits represent relevant sources of bioactive compounds with potential mechanistic relevance to PD.

These fruits and seed from the Amazonian Diet provide a wide spectrum of bioactive constituents, including polyphenols, anthocyanins, flavonoids, carotenoids, selenium, tocopherols, and unsaturated fatty acids, which have been extensively investigated for their antioxidant, anti-inflammatory, metabolic, and cytoprotective properties. Evidence indicates that these compounds may modulate key biological pathways involved in neurodegeneration, including oxidative stress, inflammatory signaling, and mitochondrial dysfunction [56,187,188,189].

**Figure 4:**
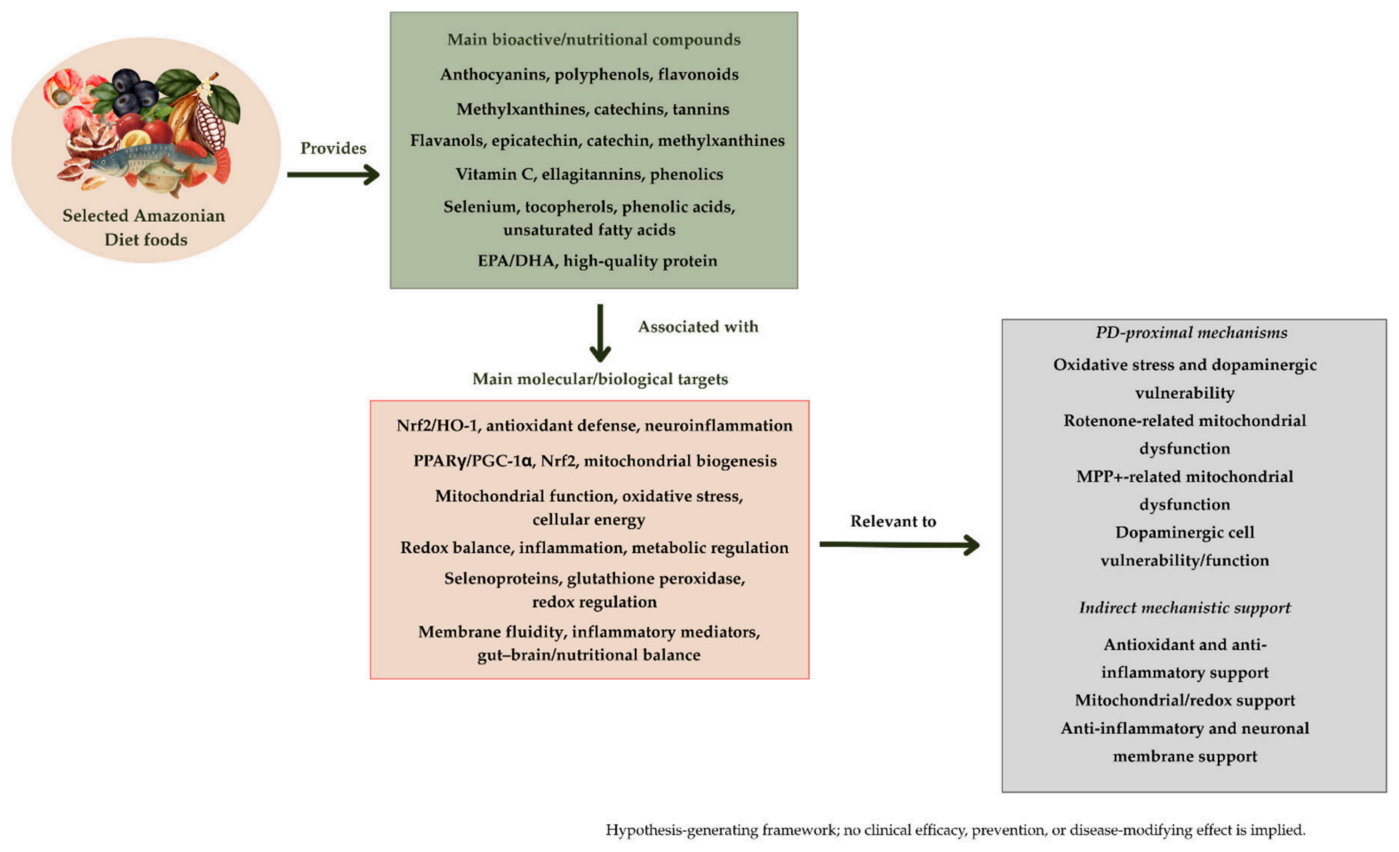
Graphical summary of the proposed relationships between selected Amazonian Diet foods, major bioactive/nutritional compounds, molecular and biological targets, and Parkinson’s disease-related pathways. Selected Amazonian foods provide anthocyanins, polyphenols, flavonoids, methylxanthines, catechins, tannins, vitamin C, selenium, tocopherols, unsaturated fatty acids, EPA/DHA, and high-quality protein. These compounds are associated with biological processes such as antioxidant defense, redox regulation, mitochondrial function, neuroinflammation, metabolic regulation, membrane fluidity, gut–brain axis signaling, and proteostasis/autophagy balance. The figure distinguishes PD-proximal mechanisms, including oxidative stress, dopaminergic vulnerability, and rotenone- and MPP+-related mitochondrial dysfunction, from indirect mechanistic support related to antioxidant, anti-inflammatory, mitochondrial, redox, and neuronal membrane pathways. This framework is hypothesis-generating and does not imply clinical efficacy, prevention, or disease-modifying effects in Parkinson’s disease. The colors of the boxes are used only for visual differentiation and have no statistical or analytical meaning, hierarchical relationships, or differences in the strength of evidence.

To integrate the evidence discussed above, Figure 4 summarizes the proposed relationships between selected Amazonian Diet foods, their main bioactive and nutritional compounds, molecular and biological targets, and their potential relevance to PD-related pathways.

### 3.7. Açaí (Euterpe oleracea) in the Amazonian Diet and Neurodegenerative Pathways

*Açaí* (*Euterpe oleracea* Mart.; *Euterpe precatoria*) is one of the most emblematic fruits of the Amazon basin and a central component of traditional dietary practices within the Amazonian Diet, where it is predominantly consumed as minimally processed pulp and integrated into long-term dietary patterns rooted in regional biodiversity [12,21]. Among Amazonian fruits, species of the genus *Euterpe* are also the most extensively investigated in experimental models of neurodegeneration, particularly in studies addressing oxidative stress, neuroinflammation, and aging-related mechanisms [190,191].

Acai berry pulp is characterized by high concentrations of anthocyanins—particularly cyanidin and malvidin derivatives—along with flavonoids, phenolic acids, and proanthocyanidins, placing it among the richest sources of polyphenols reported in edible fruits [15,19,21]. These compounds confer strong antioxidant capacity and have been widely investigated in redox-sensitive biological systems.

Evidence indicates that acai berry-derived polyphenols interact with molecular pathways implicated in neurodegenerative processes. In vitro studies demonstrate that anthocyanin-rich açaí extracts attenuate inflammatory signaling in microglial cells [81] and reduce neuroinflammatory activation in lipopolysaccharide-stimulated microglia [192]. Additional studies have shown that acai berry extracts restore calcium homeostasis and improve autophagic function in neuronal models [193], processes closely related to protein homeostasis and neuronal resilience. More recently, ALNasser and cols (2023) reported that acai berry extracts attenuate mitochondrial dysfunction and cellular redox imbalance in a glutamate-induced neurotoxicity model, supporting interactions with bioenergetic pathways relevant to Parkinson’s disease (PD) [194].

Animal studies further reinforce these findings. In MPTP-induced mouse models of PD, dietary supplementation with açaí increased Nrf2 and HO-1 expression, reduced oxidative damage, preserved dopaminergic neurons, and improved motor performance [195,196].

Complementary studies in aged rodents demonstrated that polyphenol-rich acai pulp improved cognitive performance and attenuated inflammatory signaling [192], alongside modulation of oxidative stress markers and activation of Nrf2-related pathways in brain regions [194]. In models of systemic metabolic or toxic stress, açaí supplementation reduced brain oxidative damage [86] and mitigated metal-induced oxidative stress in primary astrocyte cultures [87], suggesting broader neuroprotective potential.

Evidence from invertebrate models contributes additional insight into aging-related mechanisms. Anthocyanin-rich extracts from *Euterpe* species increased stress resistance and delayed aging-related markers in *Caenorhabditis elegans* [197] and improved survival under high-fat dietary conditions in *Drosophila melanogaster* [91]. Although these models do not permit direct clinical extrapolation, they indicate modulation of conserved pathways linked to oxidative balance, metabolic regulation, and longevity.

Current evidence does not establish a direct preventive or therapeutic role for acai in Parkinson’s disease. However, its anthocyanin-rich profile and consistent effects on oxidative stress, neuroinflammation, autophagy, mitochondrial function, and redox signaling converge with key biological processes implicated in dopaminergic degeneration. Within this context of the Amazonian Diet, regular acai berry consumption may therefore represent one component of a broader biodiversity-based dietary pattern enriched in bioactive compounds capable of modulating pathways relevant to brain aging and neurodegenerative susceptibility. Further translational and longitudinal studies in human populations are required to clarify these associations.

### 3.8. Brazil Nuts (Bertholletia excelsa) in the Amazonian Diet and Neurodegenerative Pathways

Brazil nuts (*Bertholletia excelsa*) are nutritionally distinctive within Amazonian biodiversity-based diets due to their exceptionally high selenium content, primarily in the form of selenomethionine. This trace element is essential for the synthesis and activity of selenoproteins, particularly glutathione peroxidases, which play central roles in antioxidant defense and redox homeostasis [198]. Human intervention studies have demonstrated that dietary intake of Brazil nuts effectively increases selenium status and enhances glutathione peroxidase activity in different populations, including patients undergoing hemodialysis, supporting the biological relevance of this food as a functional selenium source [107,199].

Beyond redox regulation, selenium status has been increasingly investigated in the context of cognitive decline and neurodegenerative diseases. A systematic review and meta-analysis reported associations between selenium levels and cognitive outcomes in individuals with mild cognitive impairment and Alzheimer’s disease, suggesting that adequate selenium intake may be linked to improved antioxidant capacity and neuronal resilience [192,199]. Although these findings do not directly address PD, they provide indirect clinical support for the involvement of selenium-dependent antioxidant pathways in mechanisms associated with neurodegeneration, including oxidative stress and neuronal vulnerability.

Importantly, the relationship between selenium and neurological health appears to lie within a relatively narrow optimal range, as both deficiency and excessive intake may be harmful. This reflects its tightly regulated biological role, particularly in selenoproteins involved in antioxidant defense and redox balance, such as glutathione peroxidases and thioredoxin reductases. While low selenium levels may compromise cellular protection against oxidative stress, excessive intake has been associated with pro-oxidant effects and potential neurotoxicity [200,201].

In addition to selenium, Brazil nuts are characterized by a lipid-rich matrix (approximately 60–70% of total composition), predominantly composed of unsaturated fatty acids, alongside a moderate protein content (~14–20%) [202].

Consistent with the observations from Step 1, these lipids may influence neuronal membrane composition, inflammatory signaling, and metabolic pathways implicated in neurodegeneration, further supporting the role of Brazil nuts within dietary patterns relevant to Parkinson’s disease.

### 3.9. Camu-Camu (Myrciaria dubia) in the Amazonian Diet and Neurodegenerative Pathways

Camu-camu (*Myrciaria dubia*) is a native Amazonian fruit traditionally consumed in riverine communities and increasingly recognized for its exceptionally high vitamin C content, in addition to ellagic acid, anthocyanins, and other polyphenols. Within the Amazonian Diet, camu-camu represents a micronutrient-dense component that contributes to the overall redox-modulating profile of the regional dietary pattern [110].

A systematic review by Langley et al. (2015) highlighted the remarkable antioxidant capacity of camu-camu compared to many other fruits, largely attributed to the synergistic interaction between vitamin C and polyphenolic compounds [203]. Experimental studies further support its biological activity. In vitro, camu-camu extract reduced oxidative stress and inflammatory responses through modulation of Nrf2 and NFAT signaling pathways [112], mechanisms closely associated with cellular antioxidant defense and inflammatory regulation. Earlier work also demonstrated anti-inflammatory and antioxidative effects in human models [204], reinforcing the fruit’s biological plausibility in modulating systemic oxidative balance.

Although direct studies evaluating camu-camu in PD models remain limited, the pathways influenced by its bioactive compounds intersect with central mechanisms implicated in PD pathophysiology, including oxidative stress and chronic neuroinflammation [108,110,112]. Given that dopaminergic neurons in the substantia nigra are particularly vulnerable to oxidative damage, dietary components capable of supporting endogenous antioxidant systems may be relevant within a broader neuroprotective framework [1].

A recent review of Amazonian fruits and non-communicable diseases emphasized the potential contribution of camu-camu to cardiometabolic and inflammatory regulation [186], suggesting that its effects may extend beyond isolated antioxidant activity and involve systemic metabolic pathways. Considering the interplay between metabolic dysfunction, inflammation, and neurodegenerative vulnerability, such systemic effects may hold indirect relevance for brain aging and PD-related processes.

At present, evidence does not support a specific preventive or therapeutic role for camu-camu in Parkinson’s disease. However, its high antioxidant density and demonstrated modulation of redox and inflammatory pathways provide biological plausibility that its regular consumption, as part of the Amazonian Diet pattern, may interact with mechanisms involved in aging and neurodegenerative susceptibility. Further experimental and clinical investigations are necessary to clarify its role in neurodegenerative contexts.

### 3.10. Cocoa (Theobroma cacao) in the Amazonian Diet and Neurodegenerative Pathways

Cocoa (*Theobroma cacao* L.), a species with Amazonian origin, represents an important source of flavanols—particularly epicatechin and catechin—as well as theobromine and other bioactive compounds. Within the framework of the Amazonian Diet, cocoa can be considered part of a biodiversity-based dietary pattern naturally enriched in polyphenols with recognized antioxidant and vasomodulatory properties [90,205].

Experimental evidence suggests that cocoa-derived compounds may interact with biological pathways relevant to PD, particularly those related to oxidative stress, mitochondrial function, and neuroinflammation. In a cellular model of dopaminergic toxicity, cocoa components enhanced mitochondrial biogenesis through activation of the PPARγ/PGC-1α signaling axis [206], supporting a potential role in bioenergetic regulation. In an experimental PD model, Azzolin et al. (2025) reported neuroprotective and immunomodulatory effects of a formulation combining cocoa seed husk and guaraná extract; however, the combined nature of the intervention limits attribution of these effects to cocoa alone [205].

Beyond PD-specific models, cocoa flavanols have been consistently associated with mechanisms related to brain aging and cognitive resilience. In animal studies, high-phenolic cocoa intake increased hippocampal BDNF expression and markers of neurogenesis [207]. Extracts from cocoa have been shown to directly bind with the α-synuclein protein, inhibiting the toxic aggregation that drives PD pathology [208]. In older adults, the Cocoa, Cognition, and Aging (CoCoA) Study reported improvements in cognitive performance and vascular function following cocoa flavanol consumption [116]. More recently, data from the COcoa Supplement and Multivitamin Outcomes Study (COSMOS) suggested associations between cocoa extract supplementation and biomarkers linked to inflammaging [117]. These findings highlight overlap between cocoa bioactivity and molecular pathways implicated in neurodegeneration, including oxidative imbalance, inflammatory signaling, and vascular dysfunction.

The experimental, aging-related, and mechanistic data provides biological plausibility that regular cocoa consumption—particularly within traditional and biodiversity-based dietary patterns—may interact with processes involved in neurodegenerative vulnerability. Within the Amazonian Diet, cocoa therefore represents one of several plant-derived components warranting further investigation in the context of Parkinson’s disease.

### 3.11. Guaraná (Paullinia cupana) in the Amazonian Diet and Neurodegenerative Pathways

Guaraná (*Paullinia cupana*) constitutes a relevant component of biodiversity-based Amazonian dietary patterns. Native to the Amazon basin, its seeds have been traditionally consumed in regional diets, particularly among riverine populations, as part of the Amazonian Diet rather than as an isolated stimulant. Guaraná seeds are characterized by high levels of methylxanthines—most notably caffeine—as well as substantial concentrations of catechins, epicatechins, proanthocyanidins, and other flavonoids [6,11,16].

While epidemiological studies have consistently reported inverse associations between caffeine intake and PD risk, accumulating experimental evidence indicates that the biological effects of guaraná are not solely attributable to caffeine [209]. Polyphenol-rich, caffeine-free fractions of guaraná extracts demonstrate antioxidant, cytoprotective, and mitochondrial-protective effects in dopaminergic cell models, supporting a broader spectrum of bioactivity mediated by combined methylxanthine and polyphenol mechanisms [210].

In vitro studies using SH-SY5Y dopaminergic cells exposed to rotenone have shown that guaraná extracts preserve mitochondrial integrity, attenuate oxidative damage, and reduce apoptotic markers [144]. These findings align with key pathogenic mechanisms implicated in PD, particularly mitochondrial dysfunction and oxidative stress, and suggest that guaraná-derived compounds may interact with these pathways at a cellular level.

Population-based evidence further supports the biological plausibility of systemic effects. In an elderly Amazonian study, habitual guaraná consumption was associated with a lower prevalence of metabolic morbidities and reduced biomarkers of oxidative stress [211]. Although these observational data were not designed to evaluate neurodegenerative outcomes, they indicate that long-term guaraná intake may influence metabolic and redox homeostasis—processes increasingly linked to brain aging and neurodegenerative vulnerability.

Experimental studies in invertebrate models provide additional mechanistic insight. Guaraná extracts enhanced resistance to oxidative stress and were associated with lifespan extension in Caenorhabditis elegans [212,213]. More recently, a Drosophila melanogaster model contributed to the characterization of molecular pathways potentially modulated by guaraná seed components, including conserved stress-response and metabolic signaling networks [214]. While such models do not permit direct clinical extrapolation, they reinforce the hypothesis that guaraná may interact with evolutionarily conserved mechanisms of cellular resilience.

In a toxin-induced experimental model of PD, a formulation combining guaraná extract with cocoa seed husk demonstrated neuroprotective and immunomodulatory effects associated with redox balance and inflammatory regulation [206]. However, the combined nature of the intervention precludes attribution of the observed effects specifically to guaraná, highlighting the need for caution in interpreting these findings.

Nevertheless, its biochemical composition, long-standing dietary use, and emerging experimental data provide biological plausibility that regular consumption within a biodiversity-based Amazonian dietary pattern may interact with pathways related to oxidative stress, metabolic regulation, and neurodegenerative susceptibility, particularly through the combined effects of methylxanthines and polyphenols on redox-sensitive and inflammatory mechanisms. Further translational and longitudinal studies are required to clarify these associations and their relevance to PD.

### 3.12. Relevance of Evidence to Parkinson’s Disease-Specific Mechanisms

A central consideration in the interpretation of the present findings is that several mechanisms discussed throughout this review, including oxidative stress, neuroinflammation, mitochondrial dysfunction, impaired autophagy, apoptosis, and metabolic dysregulation, are not exclusive to PD. These biological processes are shared across several neurodegenerative disorders, including Alzheimer’s disease, Huntington’s disease, amyotrophic lateral sclerosis, and other aging-related neurological conditions [215,216,217]. Therefore, antioxidant, anti-inflammatory, or metabolic effects alone were not interpreted as evidence of Parkinson’s disease-specific activity.

In this review, evidence was considered closer to Parkinson’s disease-specific relevance when it involved experimental PD models or mechanisms directly connected to established PD pathology, including dopaminergic neuronal vulnerability, nigrostriatal degeneration, mitochondrial dysfunction, impaired proteostasis/autophagy, α-synuclein aggregation-related pathways, or toxin-induced models such as MPTP, 6-OHDA, rotenone, MPP+, or paraquat [73,81,141,186,187,189,192,193,195,205,206,215,218]. In contrast, studies addressing only general antioxidant, anti-inflammatory, metabolic, cardiovascular, cognitive, or non-PD neurological outcomes were interpreted as indirect mechanistic support.

Within this framework, the degree of proximity to PD-specific evidence differed among the selected Amazonian foods. Açaí, guaraná, and cocoa/cacao presented the closest relationship with PD-relevant experimental evidence, including toxin-induced models, dopaminergic cellular vulnerability, mitochondrial dysfunction, or cells derived from patients with PD. For example, açaí has been investigated in experimental PD-related models involving dopaminergic neuroprotection and activation of the Nrf2/HO-1 pathway [73,192]; guaraná has been evaluated in rotenone-exposed SH-SY5Y dopaminergic cells, demonstrating protection against mitochondrial dysfunction and oxidative stress [144]; and cocoa/cacao has been studied in MPP+-intoxicated SH-SY5Y cells and in formulations combining cocoa seed husk and guaraná, showing effects on pathways relevant to PD pathophysiology [186].

In contrast, evidence for camu-camu and Brazil nuts remains predominantly indirect and is based mainly on compositional, antioxidant, anti-inflammatory, metabolic, mitochondrial, or redox-related mechanisms [129,130,132]. These foods therefore contribute to the broader biological plausibility of the Amazonian Diet framework but should not be interpreted as having direct PD-specific evidence.

This distinction is essential because the present review does not propose the Amazonian Diet as a therapeutic or disease-modifying intervention for Parkinson’s disease. Rather, it identifies foods and bioactive compounds whose nutritional and mechanistic profiles intersect with biological pathways implicated in PD pathophysiology, including mitochondrial dysfunction, oxidative stress, neuroinflammation, impaired autophagy, and α-synuclein-related processes [213,215]. Accordingly, the current evidence supports hypothesis generation and prioritization for future research, but does not establish clinical efficacy, prevention, or disease modification in Parkinson’s disease.

### 3.13. Integrative Perspective: The Amazonian Dietary Pattern and Parkinson’s Disease

The Amazonian Diet, as described in ethnonutritional, cultural, and epidemiological contexts, represents a biodiversity-based dietary pattern characterized by the sustained consumption of native fruits, seeds, roots, flours, and freshwater fish, predominantly in minimally processed forms This pattern is naturally enriched in polyphenols, anthocyanins, methylxanthines, carotenoids, vitamin C, selenium, and unsaturated fatty acids, and is traditionally embedded within long-term dietary practices shaped by ecological availability and cultural continuity [13,15,17]. The Amazonian Diet should be understood as a coherent food system rather than a collection of isolated functional ingredients.

Neurodegeneration is increasingly understood as a multifactorial and systemic process influenced by aging, metabolic status, vascular function, gut–brain interactions, and chronic low-grade inflammation [20,219,220]. Evidence supports the role of dietary patterns in modulating the risk and progression of Parkinson’s disease (PD), shifting the focus from isolated nutrients to integrated dietary models, as is the case in the Mediterranean, DASH and MIND diets [8,221,222,223].

Available meta-analyses of observational studies suggest that greater adherence to Mediterranean-type or healthy dietary patterns is generally associated with lower PD risk, whereas Western-type dietary patterns are associated with higher PD risk. However, recent evidence also indicates that caution is needed when interpreting healthy dietary patterns in relation to PD risk. Chen et al. reported that adherence to several healthy dietary patterns was not significantly associated with PD risk in two large prospective cohorts, suggesting that dietary patterns associated with lower risk of chronic diseases may not necessarily translate into measurable PD prevention [12]. The findings of Wang et al. further support a broader interpretation of diet as a modulator of systemic biological pathways. In large prospective cohorts, the authors identified healthy dietary patterns associated with lower risk of major chronic diseases and developed empirical dietary patterns related to inflammatory potential and insulin response [13].

To contextualize the Amazonian Diet in relation to dietary patterns more extensively studied in neurodegeneration, Table 5 compares the Amazonian, Mediterranean, and MIND diets, emphasizing their main food components, characteristic bioactive compounds, potential PD-relevant mechanisms, and relative level of human evidence.

The Amazonian, Mediterranean, DASH and MIND diets differ in geographic origin, cultural context, food availability, ecological structure, and level of scientific validation. Moreover, these established dietary models are characterized not only by the inclusion of potentially protective food groups, such as fruits, vegetables, legumes, nuts, whole grains, fish, fiber, and unsaturated lipids, but also by the restriction or negative scoring of food groups commonly associated with Western dietary patterns, including red and processed meats, fried foods, sweets, refined products, saturated fats, and ultra-processed foods [13,19].

In this context, the Amazonian Diet should be interpreted as a culturally specific and biodiversity-based dietary framework that may share selected mechanistic features with Mediterranean and MIND dietary patterns, while differing substantially in food composition, traditional use, and current level of epidemiological validation.

Therefore, the Amazonian Diet, by virtue of its biodiversity-based composition and long-standing traditional use, provides a relevant model for examining how nutrition interfaces with mechanisms of brain aging and neurodegenerative susceptibility. Thus, the potential relevance of the Amazonian Diet may depend not only on the presence of bioactive-rich regional foods, but also on the extent to which traditional dietary patterns displace Westernized food groups associated with higher inflammatory, metabolic, and oxidative burden.

Despite this, the comparison highlights that although the Amazonian Diet shares several mechanistic features with Mediterranean and MIND dietary patterns, direct human evidence in Parkinson’s disease remains insufficient.

Thus, this comparison is conceptual rather than directly comparative and should be viewed as a basis for future research, not as evidence of equivalent clinical efficacy or disease-modifying effects.

An additional aspect that should be considered when interpreting Amazonian dietary patterns is the marked seasonal variability of plant-derived foods. Unlike standardized dietary models, the availability of native fruits in Amazonian regions is strongly influenced by ecological cycles, resulting in fluctuating patterns of nutrient and bioactive compound intake throughout the year. This temporal variability may affect cumulative exposure to neuroprotective compounds and should be taken into account when interpreting both experimental findings and observational dietary data. In this context, the Amazonian Diet is better understood as a dynamic and seasonally modulated dietary system rather than a fixed nutritional pattern [53,54,67].

Nevertheless, the current body of evidence does not support causal or disease-modifying claims regarding the Amazonian Diet in Parkinson’s disease. Most available data derive from in vitro systems, animal models, and observational studies not specifically designed to assess PD-related outcomes. In addition, environmental and ecological factors—including food preparation practices, sustainability, and potential exposure to environmental contaminants—should be considered when interpreting findings derived from traditional dietary systems.

The multivariate analyses identified distinct clusters of Amazonian Diet foods characterized by integrated nutritional and bioactive profiles, supporting the selection of specific fruits and seeds for mechanistic exploration. Acai berry, Brazil nuts, camu-camu, cocoa, and guaraná were identified based on their integrated nutritional and bioactive profiles. Evidence derived from experimental models and mechanistic studies indicates that compounds present in these foods interact with biological pathways implicated in PD, including oxidative stress, mitochondrial dysfunction, neuroinflammation, impaired autophagy, and metabolic dysregulation. Importantly, these effects appear to emerge from the combined action of multiple compounds within whole foods, rather than from isolated nutrients.

These findings, supported by integrated compositional and clustering analyses, reinforce the relevance of biodiversity-based dietary patterns as a framework for exploring diet–neurodegeneration interactions. Taken together, the Amazonian Diet should not be interpreted as a therapeutic intervention for Parkinson’s disease. Rather, it represents a biologically plausible and hypothesis-generating nutritional framework that expands dietary pattern research beyond conventional models.

Although several Amazonian foods and bioactive compounds discussed in this review have been associated with antioxidant, anti-inflammatory, mitochondrial, metabolic, or neuroprotective properties, these findings should be interpreted with caution. Most available studies were conducted using isolated compounds, extracts, in vitro systems, or animal models, and many did not directly evaluate Parkinson’s disease-specific outcomes. Therefore, the evidence summarized here does not support therapeutic claims, clinical recommendations, disease prevention, or disease modification in Parkinson’s disease. Instead, it provides a hypothesis-generating framework suggesting that selected Amazonian foods may interact with biological pathways implicated in PD pathophysiology and should be further investigated in human studies using realistic dietary exposures and whole-food matrices.

### 3.14. Translational Considerations: Bioavailability, Blood–Brain Barrier Penetration, and Dietary Exposure

The potential relevance of Amazonian Diet foods to Parkinson’s disease (PD) depends not only on their bioactive composition but also on bioavailability, metabolism, tissue distribution, and realistic dietary exposure. Phenolic compounds, anthocyanins, flavonoids, carotenoids, methylxanthines, and selenium-containing compounds undergo digestion, intestinal transformation, hepatic metabolism, and microbiota-mediated bioconversion before reaching target tissues [20,21,30,36]. These processes influence their biological activity and should be considered when interpreting experimental and clinical evidence.

In this context, foods such as açaí, cocoa/cacao, guaraná, camu-camu, and Brazil nuts may act through complementary mechanisms. Some compounds or metabolites may reach the circulation and, in specific cases, interact with the central nervous system, whereas others may exert indirect effects through systemic inflammation, oxidative balance, mitochondrial metabolism, vascular function, gut barrier integrity, and microbiota-derived metabolites [20,58,59]. These pathways are relevant because PD is increasingly recognized as a multisystem disorder involving dopaminergic neurodegeneration, gastrointestinal dysfunction, immune alterations, microbiota changes, and gut–brain axis signaling [17,18,28].

The ability of bioactive compounds to reach the brain varies according to their chemical structure and metabolic fate. Methylxanthines such as caffeine are associated with neurocognitive and neurological effects and may be relevant in the context of guaraná and other caffeine-containing foods [40]. Many phenolic compounds exert biological effects mainly through metabolites involved in inflammatory, oxidative, and energy metabolism pathways [21]. Carotenoids and other lipid-soluble compounds may contribute to neuroprotective mechanisms, although their effects depend on matrix composition, digestion, and transport [36]. Selenium and other trace elements may also be relevant to PD-related redox pathways, and Brazil nuts have been associated with improvements in oxidative stress and inflammatory biomarkers in clinical contexts [30,102]. Together, these factors suggest that Amazonian foods may influence PD-related pathways through both direct and indirect mechanisms.

However, translation to dietary recommendations remains limited. Many experimental studies use isolated compounds, concentrated extracts, or model systems that may not reflect habitual dietary intake [48,49]. In contrast, the Amazonian Diet involves regular consumption of whole foods with complex combinations of bioactives, fiber, lipids, micronutrients, and other phytochemicals, which may favor synergistic and long-term physiological effects [15,23,24]. Therefore, the potential role of phenolic-rich Amazonian foods in PD should be interpreted in light of realistic intake, bioavailability, metabolism, and food-matrix interactions. Future studies should prioritize habitual consumption levels, processing effects, seasonal variability, circulating metabolites, gut–brain axis markers, and clinically relevant outcomes.

## 4. Limitations

Some limitations should be considered when interpreting these findings. First, this study was designed as an exploratory and hypothesis-generating integrative review with multivariate analysis, rather than as a systematic review or meta-analysis. Therefore, the results should not be interpreted as evidence that the Amazonian Diet prevents Parkinson’s disease (PD), modifies disease progression, improves symptoms, alters levodopa response, or affects clinical biomarkers. Rather, this work examines whether selected Amazonian foods contain nutritional and bioactive components that intersect with biological pathways implicated in PD pathophysiology.

Second, the evidence base remains heterogeneous and fragmented. The available studies differ in food matrix, biological model, experimental design, analytical methods, dose or exposure, and outcomes assessed. Most evidence refers to isolated foods, extracts, or compounds, with limited data on the Amazonian Diet as an integrated dietary pattern. Robust human clinical studies directly evaluating Amazonian foods or dietary patterns in PD are still lacking.

Third, variability in the characterization of bioactive compounds limits quantitative comparisons across studies. Because extraction, identification, and quantification methods are not standardized, this review adopted a qualitative, presence-based approach to assess bioactive richness. Although useful for comparing heterogeneous sources, this strategy does not capture absolute concentrations, bioavailability, processing effects, seasonal variation, or food-matrix interactions.

Fourth, the clustering analysis reflects only the variables included in the analytical matrix: dietary fiber, protein content, vitamin richness, mineral richness, polyphenol richness, carotenoid richness, and miscellaneous bioactive richness. Thus, the observed clusters should be interpreted as exploratory patterns derived from this specific coding strategy. The inclusion of additional variables, such as detailed fatty acid profiles, amino acid composition, or micronutrient concentrations, could result in different grouping patterns.

Fifth, although Amazonian fish are important components of the regional diet and may contribute protein, EPA, DHA, and other polyunsaturated fatty acids, their relevance to PD remains indirect. In this study, fish-related interpretations were based mainly on compositional data and broader mechanistic evidence regarding omega-3 fatty acids, neuroinflammation, membrane function, mitochondrial regulation, and the gut–brain axis. No included study directly evaluated Amazonian fish intake in PD models or clinical PD outcomes. Regarding vitamin D, available compositional data for Amazonian freshwater fish remain limited. In the Brazilian Food Composition Table, vitamin D values are frequently unavailable for the edible muscle or fillet of Amazonian fish species, including pirarucu. Therefore, vitamin D was not emphasized as a central nutrient in the present analysis, and future studies should quantify vitamin D in edible portions of Amazonian fish species using standardized analytical methods.

Sixth, studies on Amazonian dietary patterns vary in population characteristics, dietary assessment methods, regional food availability, socioeconomic context, healthcare access, and potential underreporting of PD. These studies provide valuable contextual information on food selection and dietary representativeness, but they were not designed to assess PD incidence, progression, treatment response, or neuroprotective effects.

Finally, comparisons between the Amazonian Diet and established dietary models, such as the Mediterranean and MIND diets, are conceptual rather than directly comparative. These dietary patterns differ in geographic origin, cultural context, food availability, ecological structure, and level of scientific validation. Therefore, the parallels proposed here should be viewed as a basis for future research, not as evidence of equivalent clinical efficacy.

Despite these limitations, integrating compositional data, multivariate analysis, and mechanistic evidence provides a biologically plausible framework for generating hypotheses about Amazonian dietary components and PD-related pathways. Future studies should prioritize standardized food composition analyses, longitudinal dietary assessments, controlled clinical trials, and mechanistic investigations of whole-food matrices and dietary patterns in populations at risk for, or living with, PD.

## 5. Conclusions

This review was structured around the conceptual triad Amazonian Diet–Neuroprotection–Parkinson’s Disease, proposing a biodiversity-based dietary system as a framework for hypothesis generation in neurodegenerative research. Rather than focusing on isolated nutrients, the Amazonian Diet is understood as a culturally embedded and ecologically sustained dietary pattern characterized by long-term exposure to complex phytochemical matrices derived from native fruits, seeds, freshwater fish, and minimally processed plant foods.

Across experimental and preclinical models, key components of this dietary pattern—including açaí, guaraná, cocoa, camu-camu, and Brazil nuts—have been shown to interact with molecular pathways implicated in Parkinson’s disease, such as oxidative stress, mitochondrial dysfunction, neuroinflammation, and loss of neuronal resilience. While current evidence does not support preventive or disease-modifying claims, convergent mechanistic findings provide biological plausibility within this integrative framework.

By situating neuroprotection at the interface between dietary exposure and pathological vulnerability, this work expands dietary pattern research beyond traditional Eurocentric models and highlights the relevance of cultural and ecological contexts in nutritional neuroscience. Future studies integrating epidemiological approaches, clinical investigations, and mechanistic research will be essential to determine whether biodiversity-based dietary patterns can influence neurodegenerative risk and disease trajectories.

## Figures and Tables

**Table 1:** Proximate composition of fruits, plant-based foods, and fishes (g/100 g) commonly consumed in the Amazonian Diet by riparian communities.

Common Name	Brazilian Name	Scientific Name	Energy (Kcal)	Carb (g)	Protein (g)	Lipids (g)	Fibers (g)
**Fruits/other plant-based foods**							
Abiu fruit	Abiu	*Pouteria caimito*	66	14.9	0.83	0.70	1.24
Acai berry	Açaí	*Euterpe oleracea*	68	7.66	0.90	5.01	5.89
Araza fruit	Araçá-boi	*Eugenia stipitata*	33	4.50	0.40	0.08	4.50
Bacuri fruit	Bacuri	*Platonia insignis*	52	7.54	0.49	0.58	5.20
Brazil nuts	Castanha do Brasil	*Bertholletia excelsa*	674	15.1	14.5	63.5	7.90
Buriti fruit	Buriti	*Mauritia flexuosa*	207	22.4	2.19	15.4	5.00
Camu-camu	Camu-camu	*Myrciaria dubia*	30	6.91	0.45	0.20	0.47
Cashew apple	Caju	*Anacardium occidentale*	43	10.1	0.77	0.49	2.34
Cocoa	Cacau	*Theobroma cacao*	31	7.76	0.36	0.06	2.39
Cocona	Cubiu	*Solanum sessiliflorum*	18	3.24	0.75	0.06	1.46
Cupuaçu	Cupuaçu	*Theobroma grandiflorum*	53	10.5	1.11	1.25	2.42
Guarana	Guaraná	*Paullinia cupana*	10.5	1.11	1.25	2.42	0.00
Ice-cream bean	Ingá	*Inga edulis*	52	12.5	1.1	0.2	0.00
Mangaba fruit	Mangaba	*Hancornia speciosa*	56	11.6	1.01	1.51	3.40
Murici fruit	Murici	*Byrsonima crassifolia*	90	19.3	0.88	2.58	6.80
Passion fruit	Maracujá	*Passiflora edulis*	16	1.16	0.19	0.20	0.10
Peach palm	Pupunha	*Bactris gasipaes*	212	35.4	2.59	8.34	6.39
Piquia fruit	Piquiá	*Caryocar villosum*	65	15.8	0.85	0.21	NA
Pineapple	Abacaxi	*Ananas comosus*	50	11.6	0.68	0.33	1.2
Soursop	Graviola	*Annona muricata*	66	16.8	1.0	0.3	1.24
Tucuma fruit	Tucumã	*Astrocaryum aculeatum*	262	26.5	2.03	19.1	12.7
Yellow mombin	Cajá, taperebá	*Spondias mombin*	40	9.90	0.60	0.20	4.68
Cassava	Mandioca	*Manihot esculenta*	125	30.1	1.00	0.40	6.08
Jambu leaf	Jambu	*Acmella oleracea*	121	0	20.5	4.1	2.20
**Fishes**							
Amazonian croaker	Pescada	*Plagioscion* spp.	118	0	22.0	2.5	
Amazonian sardine	Sardinha amazônica	*Triportheus* spp.	121	0	20.5	4.1	
Armored catfish	Bodó	*Hypostomus* spp.	110	0	19.3	3.2	
Curimatã	Curimatã	*Prochilodus nigricans*	123	0	21.0	3.8	
Jaraqui	Jaraqui	*Semaprochilodus* spp.	125	0	20.8	4.0	
Mapará	Mapará	*Hypophthalmus* spp.	180	0	19.5	11.0	
Matrinxã	Matrinxã	*Brycon amazonicus*	135	0	21.5	5.2	
Pacu	Pacu	*Piaractus* spp.	160	0	20.9	8.2	
Peacock bass	Tucunaré	*Cichla* spp.	121	0	24.9	2.3	
Pirapitinga	Pirapitinga	*Piaractus brachypomus*	150	0	21.0	7.0	
Pirarucu	Pirarucu	*Arapaima gigas*	130	0	22.5	4.3	
Tambaqui	Tambaqui	*Colossoma macropomum*	140	0	22.0	6.5	

**Table 2:** Protein content of major food groups in the Amazonian Diet and considerations for levodopa therapy in Parkinson’s disease.

Food Group	Examples	Typical Protein (g/100 g)	Considerations During Daytime Levodopa Therapy	General Pharmacokinetic Consideration
Amazonian fruits	Abiu, Acai berry, Araza, Bacuri, Buriti, Camu-camu, Cubiu, Cupuaçu, Ice cream bean, Mangaba, Murici, Passion fruit, Pineapple, Piquiá, Tucumã, Yellow mombin	<2 g	Generally compatible with daytime levodopa intake due to low protein content	Usually compatible with daytime levodopa intake
Fruit seeds and nuts	Brazil nuts, Cacao	14–20 g	Moderate portions may be advisable during daytime levodopa therapy	Usually compatible with evening time levodopa intake
Amazonian freshwater fish	Sardine, Bodó, Curimatã, Jaraqui, Mapará, Matrinxã, Pirapitinga, Pacu, Pescada, Pirarucu, Tambaqui, Tucunaré	20–26 g	Larger protein loads may compete with levodopa absorption	Larger protein portions may require individualized timing
Tubers and vegetables	Cassava and Jambu	<3 g	Low protein content generally compatible with daytime intake	Usually compatible with daytime levodopa intake

Data were obtained primarily from the Brazilian Food Composition Database (TBCA). Levodopa shares intestinal and blood–brain barrier transport mechanisms with large neutral amino acids (LNAAs), including leucine, isoleucine, valine, phenylalanine, and tyrosine. These considerations are extrapolated from known levodopa–LNAA pharmacokinetics and should not be interpreted as specific clinical recommendations for the Amazonian Diet. Protein timing should be individualized, particularly in older adults with PD at risk of sarcopenia, frailty, or inadequate protein intake.

**Table 5:** Comparative overview of the Amazonian Diet, Mediterranean Diet, and MIND Diet in relation to neuroprotective nutritional features and potential relevance to Parkinson’s disease.

Feature	Amazonian Diet	Mediterranean Diet	MIND Diet
Cultural and geographical context	Biodiversity-based dietary pattern from Amazonian riverine and traditional food systems, characterized by native fruits, seeds, freshwater fish, and cassava-derived foods.	Traditional dietary pattern from Mediterranean regions, characterized by olive oil, fruits, vegetables, legumes, whole grains, nuts, herbs, and fish/seafood.	Hybrid dietary pattern derived from Mediterranean and DASH diets, developed specifically to support brain health and reduce cognitive decline.
Main plant-based foods	Açaí, camu-camu, guaraná, cocoa/cacao, Brazil nuts, buriti, tucumã, peach palm, cassava-derived foods, and other native fruits.	Grapes, citrus fruits, apples, pears, figs, pomegranates, tomatoes, leafy vegetables, legumes, nuts, herbs, and whole grains.	Berries, especially blueberries and strawberries, green leafy vegetables, other vegetables, nuts, beans, whole grains, and olive oil.
Main lipid and protein sources	Amazonian freshwater fish, Brazil nuts, palm fruits, and other lipid-rich native foods; fish provides high-quality protein and may contribute EPA/DHA depending on species.	Extra-virgin olive oil, nuts, seeds, legumes, and oily marine fish rich in unsaturated fatty acids and long-chain omega-3 fatty acids.	Olive oil, nuts, legumes, whole grains, and fish; emphasizes food-group frequency rather than specific species or regional foods.
Characteristic bioactive compounds	Polyphenols and anthocyanins; flavanoids, catechins, epicatechins, and methylxanthines; vitamin C and ellagitannin-related phenolics; selenium, tocopherols, phenolic acids, and unsaturated fatty acids; carotenoids.	Olive oil phenolics, resveratrol, anthocyanins, flavonoids, carotenoids, tocopherols, phytosterols, fiber, and omega-3 fatty acids from fish.	Anthocyanins from berries, lutein/zeaxanthin and folate from leafy greens, vitamin E and unsaturated fatty acids from nuts and olive oil, and fiber from whole grains and legumes.
Potential mechanisms relevant to PD	Biological plausibility through modulation of oxidative stress, neuroinflammation, mitochondrial function, gut–brain axis signaling, redox balance, and metabolic regulation.	Supported by evidence involving oxidative stress, neuroinflammation, mitochondrial function, vascular health, metabolic regulation, gut microbiota, and aging-related neurodegeneration.	Mainly linked to cognitive protection, reduced neuroinflammation, vascular and metabolic support, antioxidant intake, and brain-aging pathways.
Human evidence in PD or neurological outcomes	No clinical trial or prospective cohort study was identified evaluating the Amazonian Diet as a whole dietary pattern in relation to PD incidence, progression, symptoms, levodopa response, or biomarkers. Evidence for individual foods remains mostly indirect.	Supported by observational studies and limited interventional evidence in PD and neurological outcomes, although robust evidence for disease modification remains limited.	Strongest evidence relates to cognitive decline and brain aging; PD-specific evidence is more limited and mostly indirect.
Evidence for individual foods/components	Açaí, cocoa/cacao, guaraná, camu-camu, and Brazil nuts have been evaluated mainly in compositional, preclinical, biomarker, metabolic, antioxidant, inflammatory, vascular, or cognitive contexts, but not as clinically validated PD interventions.	Individual components such as olive oil, nuts, fruits, vegetables, legumes, whole grains, and fish have been studied in relation to vascular, metabolic, inflammatory, cognitive, and neurodegenerative outcomes.	Food-group evidence emphasizes berries, leafy greens, nuts, whole grains, olive oil, and fish, primarily in relation to cognitive outcomes and brain aging.
Interpretation for the present review	Hypothesis-generating dietary framework with biological plausibility, but insufficient direct human evidence for PD-specific recommendations.	Useful reference model for dietary-pattern research in PD and neurodegeneration, but not directly interchangeable with the Amazonian Diet.	Useful brain-health comparator, especially for cognitive outcomes, but not a direct model of Amazonian dietary biodiversity.

PD: Parkinson’s disease; EPA: eicosapentaenoic acid; DHA: docosahexaenoic acid; DASH: Dietary Approaches to Stop Hypertension; MIND: Mediterranean-DASH Intervention for Neurodegenerative Delay. The Amazonian Diet is presented as a hypothesis-generating dietary framework rather than as a clinically validated intervention for Parkinson’s disease. Note: Although the DASH diet is not shown separately in Table 5, it is considered conceptually because the MIND Diet incorporates elements of both the Mediterranean and DASH dietary patterns. Thus, the comparison focuses on the Mediterranean and MIND diets as the most frequently discussed neuroprotective dietary models, while acknowledging DASH as part of the conceptual basis of the MIND Diet.

## Data Availability

All data analyzed in this review are publicly available in the cited databases (TBCA—Brazilian Food Composition Table; FAO/INFOODS; PubMed/MEDLINE; ScienceDirect; SciELO). No new datasets were generated.

## Supplementary Materials

The following supporting information can be downloaded at: https://www.mdpi.com/article/10.3390/nu18152472/s1, Table S1. Search strategy and conceptual search blocks used in Stage 1 and Stage 2; Table S2. Eligibility and exclusion criteria used in the integrative evidence mapping; Table S3. Evidence classification framework used to distinguish translational strength and proximity to Parkinson’s disease; Table S4. Proximity of evidence to Parkinson’s disease-specific mechanisms; Table S5. Direct and indirect mechanistic evidence for prioritized Amazonian fruits and seeds.
