# Supplementary material for: Potential Relevance of Amazonian Diet Components in Parkinson’s Disease: An Integrative Review with Multivariate Analysis
Source: Nutrients. 2026 Jul 30;18(15):2472. doi: 10.3390/nu18152472 (PMC13468174; doi:10.3390/nu18152472)
Supplement: Supplementary file 1 [file nutrients-18-02472-s001.zip › nutrients-4368701-supplementary.pdf]

Supplementary Materials

Potential Relevance of Amazonian Diet Components in Parkinson's Disease: An Integrative Review with Multivariate Analysis

This supplementary file provides the search strategies, eligibility criteria, evidence classification framework, and the table distinguishing direct and indirect evidence used to support the integrative review.

Table S1. Search strategy and conceptual search blocks used in Stage 1 and Stage 2.

Search component	Database	Purpose	Search strategy
Search 1	PubMed/MEDLINE and SciELO	Compositional and bioactive mapping of Amazonian fruits and plant-derived foods	"Abiu" OR "Pouteria caimito" OR "Açaí" OR "Acai berry" OR "Euterpe oleracea" OR "Araza" OR "Eugenia stipitata" OR "Bacuri fruit" OR "Platonia insignis" OR "Brazil nut" OR "Bertholletia excelsa" OR "Buriti fruit" OR "Mauritia flexuosa" OR "Cocoa" OR "Theobroma cacao" OR "Cashew apple" OR "Anacardium occidentale" OR "Camu-camu" OR "Myrciaria dubia" OR "Cocona" OR "Solanum sessiliflorum" OR "Cupuaçu" OR "Theobroma grandiflorum" OR "Guarana" OR "Paullinia cupana" OR "Ice-cream bean" OR "Inga edulis" OR "Mangaba fruit" OR "Hancornia speciosa" OR "Murici fruit" OR "Byrsonima crassifolia" OR "Passion fruit" OR "Passiflora edulis" OR "Peach palm" OR "Bactris gasipaes" OR "Pineapple" OR "Ananas comosus" OR "Piquia fruit" OR "Caryocar villosum" OR "Soursop" OR "Annona muricata" OR "Yellow mombin" OR "Spondias mombin" OR "Tucuma fruit" OR "Astrocaryum aculeatum" OR "Cassava" OR "Manihot esculenta" OR "Jambu leaf" OR "Acmella oleracea".
Search 1	PubMed/MEDLINE and SciELO	Compositional and bioactive mapping of Amazonian fish	"Amazonian croaker" OR "Pescada" OR "Plagioscion spp." OR "Amazonian sardine" OR "Sardinha amazônica" OR "Triportheus spp." OR "Armored catfish" OR "Bodó" OR "Hypostomus spp." OR "Curimatã" OR "Prochilodus nigricans" OR "Jaraqui" OR "Semaprochilodus spp." OR "Mapará" OR "Hypophthalmus spp." OR "Matrinxã" OR "Brycon amazonicus" OR "Pacu" OR "Piaractus spp." OR "Peacock bass" OR "Tucunaré" OR "Cichla spp." OR "Pirapitinga" OR "Piaractus brachypomus" OR "Pirarucu" OR "Arapaima gigas" OR "Tambaqui" OR "Colossoma macropomum".
Search 1	PubMed/MEDLINE and SciELO	Direct search combining Amazonian foods and Parkinson's disease	"Parkinson's disease" AND ("nutrition" OR "antioxidants" OR "functional foods") AND ("Amazon" OR "Amazonian" OR "açaí" OR "acai" OR "camu camu" OR "Brazil nut" OR "guaraná" OR "guarana" OR "cocoa" OR "cacao").
Search 2A	PubMed/MEDLINE and SciELO	Direct search for Amazonian diet and Parkinson's disease	"Amazonian diet" AND "Parkinson's disease"; "Parkinson's disease" AND "Amazonian fruit".
Search 2B	PubMed/MEDLINE and SciELO	Prioritized fruits and PD-related mechanisms	"Parkinson's disease" AND ("nutrition" OR "diet" OR "antioxidants" OR "neuroprotection") AND ("Amazon" OR "Amazonian" OR "açaí" OR "acai" OR "guaraná" OR "guarana" OR "camu camu" OR "Brazil nut" OR "buriti" OR "cacao" OR "cocoa").
Search 2C	ScienceDirect and SciELO	Prioritized fruits and neurodegeneration-related mechanisms	"Parkinson's disease" AND ("neurodegenerative disease" OR "anti-inflammatory" OR "functional foods") AND ("Amazon" OR "Amazonian" OR "açaí" OR "acai" OR "camu camu" OR "Brazil nut" OR "guaraná" OR "guarana" OR "cocoa" OR "cacao"); "Neuroprotection" OR "Oxidative stress" OR "Inflammation" OR "Mitochondrial dysfunction" OR "Neuroinflammation" OR "Inflammaging".

Note. Search syntax was adapted to each database according to field availability and interface constraints. Search 1 was used for broad compositional and bioactive mapping, whereas Search 2 was used as a targeted complementary search to support mechanistic interpretation of prioritized foods.

Table S2. Eligibility and exclusion criteria used in the integrative evidence mapping.

Criterion	Stage	Description
Inclusion criteria	Stage 1	Studies involving selected Amazonian foods or fish species; nutritional composition; chemical composition; bioactive compounds; functional properties; antioxidant, anti-inflammatory, metabolic, neurofunctional, or neuroprotective properties potentially relevant to PD-related pathways.
Inclusion criteria	Stage 2	Studies involving the five prioritized fruits and seeds (açaí, cocoa/cacao, camu-camu, guaraná, and Brazil nuts) and mechanisms directly or indirectly related to PD, neurodegeneration, oxidative stress, neuroinflammation, mitochondrial dysfunction, dopaminergic signaling, autophagy, apoptosis, metabolic regulation, or related biological pathways.
Exclusion criteria	Both stages	Reviews, meta-analyses, or purely in silico studies were excluded from primary mapping when the objective was to identify experimental or compositional evidence; non-English studies; duplicates; studies unrelated to the selected foods after title/abstract screening; studies focused on residues, non-edible parts, supplements, cosmetics, nano-structured or encapsulated compounds, or plant breeding. Contextual reviews were retained only when used to support interpretation rather than primary evidence coding.
Screening process	Both stages	Records were screened sequentially by title, abstract, and full-text relevance. Extracted information included food or compound, study design, model, dose or exposure when available, biological outcome, PD-specific or non-specific mechanism, and evidence category.
Reviewer checking	Both stages	Extracted data were checked by a second reviewer, and disagreements were resolved by consensus.

Table S3. Evidence classification framework used to distinguish translational strength and proximity to Parkinson's disease.

Evidence type	Relative strength	Interpretation in this review
Compositional database value	Lowest translational strength	Food composition tables, proximate composition, micronutrients, fatty acids, or bioactive presence-based profiles. Used to characterize foods and support hypothesis generation; not interpreted as mechanistic or clinical evidence.
In vitro evidence	Low to moderate translational strength	Cellular models evaluating oxidative stress, inflammation, mitochondrial function, autophagy, proteostasis, dopaminergic vulnerability, or toxicity-related endpoints.
Animal toxin-model evidence	Moderate translational strength	In vivo experimental models relevant to PD or neurodegeneration, including MPTP, 6-OHDA, rotenone, paraquat, or other models of neurotoxicity or aging-related mechanisms.
Observational human evidence	Moderate translational strength	Human epidemiological or observational data evaluating habitual food intake, metabolic outcomes, inflammatory markers, cognitive or neurological outcomes, or relevant exposures.
Human clinical trial	Highest translational strength in this review framework	Intervention studies in humans evaluating food components or related bioactives. When not conducted in PD populations, these were interpreted as indirect human evidence only.

Table S4. Proximity of evidence to Parkinson's disease-specific mechanisms.

Evidence proximity category	Definition
Direct PD-related evidence	Experimental PD models, dopaminergic neuronal vulnerability, nigrostriatal degeneration, mitochondrial Complex I dysfunction, α-synuclein-related pathways, levodopa-related mechanisms, or human evidence involving PD.
Indirect neurological evidence	Evidence involving other neurological, neurodegenerative, psychiatric, cognitive, or aging-related brain outcomes without direct PD-specific models or patients.
Systemic mechanistic evidence	Evidence related to oxidative stress, systemic inflammation, metabolic regulation, vascular health, mitochondrial function, or gut–brain-relevant mechanisms without neurological specificity.
Compositional or functional-property evidence	Nutritional composition, chemical composition, antioxidant capacity, anti-inflammatory potential, or functional properties without disease-specific outcomes.

Table S5. Direct and indirect mechanistic evidence for prioritized Amazonian fruits and seeds.

Direct evidence refers to studies involving experimental PD models, dopaminergic pathways, PD-related toxicants, α-synuclein, levodopa-related mechanisms, or patients with PD. Indirect evidence refers to studies addressing general neuroprotection, antioxidant or anti-inflammatory mechanisms, other neurodegenerative conditions, systemic outcomes, or compositional evidence without PD-specific endpoints.

Food	Type	Mechanism/evidence	Subtype	Model/study	Reference
**Açaí (Euterpe oleracea Mart.)**					
Açaí	**DIRECT**	Protection against rotenone-induced cytotoxicity in vitro; reduction of oxidative stress in dopaminergic neurons	Experimental PD model	In vitro (neuronal cells + rotenone)	Machado et al. (2016)
Açaí	**DIRECT**	Dopaminergic neuroprotection through activation of Nrf2-HO-1 pathways; attenuation of PD progression	Experimental PD model	In vivo (murine PD model)	D'Amico et al. (2022)
Açaí	**DIRECT**	Attenuation of manganese-induced oxidative stress in primary rat astrocytes, relevant to toxicant-associated parkinsonism	Neurotoxicity model	In vitro (primary astrocytes)	da Silva Santos et al. (2014)
Açaí	**DIRECT**	Protection of neurons against L-glutamate toxicity; limitation of mitochondrial dysfunction and cellular redox stress	Dopaminergic neuroprotection	In vitro (neural cells)	ALNasser et al. (2023)
Açaí	INDIRECT	Modulation of oxidative stress, inflammation, and autophagy; Nrf2 expression in hippocampus and frontal cortex	Neuroinflammation / Nrf2	In vivo (rats fed açaí)	Poulose et al. (2017)
Açaí	INDIRECT	Improved cognition in aged rats; attenuation of inflammatory signaling in BV-2 microglia	Cognition / general neuroinflammation	In vivo + in vitro (aged rats / BV-2)	Carey et al. (2017)
Açaí	INDIRECT	Modulation of the NLRP3 inflammasome pathway; potential anti-neuroinflammatory activity	Neuroinflammation / NLRP3	In vitro (microglia)	Cadoná et al. (2021)
Açaí	INDIRECT	Anti-neuroinflammatory effect in LPS-activated microglia	General neuroinflammation	In vitro (LPS-stimulated microglia)	de Souza et al. (2022)
Açaí	INDIRECT	Restoration of calcium dysregulation and inhibition of autophagy in rodent brain cells	Autophagy / cellular homeostasis	In vitro (brain cells)	Poulose et al. (2014)
Açaí	INDIRECT	Antioxidant, anti-inflammatory, and anti-apoptotic effects on cognition; potential relevance to Alzheimer's disease	Neuroprotection (Alzheimer's disease)	Narrative review	Ferreira et al. (2025)
Açaí	INDIRECT	Açaí flavonoids and neurodegenerative diseases in general	General neurodegeneration	Literature review	Oliveira et al. (2019)
Açaí	INDIRECT	Neuroprotective potential of açaí and guaraná	General neuroprotection	Narrative review	Queiroz et al. (2023)
Açaí	INDIRECT	Euterpe genus and neuroprotection	General neuroprotection	Scoping review	Da Silva et al. (2023)
Açaí	INDIRECT	Improvement of inflammatory biomarkers without neurological specificity in metabolic syndrome	Systemic anti-inflammatory effect	RCT (humans with metabolic syndrome)	Kim et al. (2018)
**Brazil nuts (Bertholletia excelsa)**					
Brazil nuts	INDIRECT	Selenium supplementation and mild cognitive impairment / Alzheimer's disease	Neuroprotection (Alzheimer's disease / MCI)	Meta-analysis of RCTs	Pereira et al. (2022)
Brazil nuts	INDIRECT	Effects of regular Brazil nut consumption on general health	General health / antioxidant	Systematic review of RCTs	da Silva et al. (2022)
Brazil nuts	INDIRECT	Selenium and human health: broad review of mechanisms and clinical evidence	Micronutrient / antioxidant	Narrative review	Rayman (2012)
Brazil nuts	INDIRECT	Selenium neurotoxicity in humans; safety concern related to excessive exposure	Toxicity / safety	Epidemiological-laboratory review	Vinceti et al. (2014)
Brazil nuts	**DIRECT**	Composition of essential trace elements in PD, including selenium	Trace elements in PD	Systematic review	Popescu et al. (2025)
Brazil nuts	**DIRECT**	Copper, ceruloplasmin, zinc, and manganese in the brain and biological fluids of patients with PD	Metals and PD	Meta-analysis	Jiménez-Jiménez et al. (2026)
Brazil nuts	**DIRECT**	Common and trace metals in Alzheimer's disease and PD	Metals in PD/Alzheimer's disease	Systematic review	Doroszkiewicz et al. (2023)
Brazil nuts	INDIRECT	Anti-inflammatory activity of fatty ethanolamides from Brazil nuts and acute toxicity evaluation	Systemic anti-inflammatory effect	In vivo (zebrafish)	Quitian-Useche et al. (2021)
Brazil nuts	INDIRECT	Improvement of oxidative stress and inflammatory biomarkers in hemodialysis patients	Systemic anti-inflammatory / antioxidant effect	RCT (renal patients)	Stockler-Pinto et al. (2014)
Brazil nuts	INDIRECT	Chemical composition of selected nut seeds	Nutritional composition	Analytical study	Venkatachalam & Sathe (2006)
**Camu-camu (Myrciaria dubia)**					
Camu-camu	**DIRECT**	Neuroprotective effects of camu-camu dry residue in C. elegans	Neurodegeneration model (C. elegans)	In vivo (C. elegans)	Azevêdo et al. (2015)
Camu-camu	**DIRECT**	Modulation of neural function in Drosophila melanogaster under oxidative stress	Neurodegeneration model (Drosophila)	In vivo (Drosophila)	Musachio et al. (2024)
Camu-camu	INDIRECT	Antioxidant and anti-inflammatory activity; cardiovascular and metabolic potential	Systemic antioxidant / anti-inflammatory effect	Clinical study (humans)	Inoue et al. (2008)
Camu-camu	INDIRECT	Systematic review of antioxidant capacity and anti-inflammatory activity of camu-camu	Antioxidant / anti-inflammatory effect	Systematic review	Langley et al. (2015)
Camu-camu	INDIRECT	Antioxidant and antihypertensive compounds from camu-camu seed peel	Antioxidant / antihypertensive effect	In vitro	Fidelis et al. (2018)
Camu-camu	INDIRECT	Chemical composition and antioxidant activity of camu-camu seed extract	Composition / antioxidant activity	Analytical study	Flores Trindade et al. (2025)
Camu-camu	INDIRECT	Analgesic and anti-edematogenic activity of camu-camu juice in mice	Anti-inflammatory / analgesic effect	In vivo (mice)	da Silva et al. (2021)
Camu-camu	INDIRECT	Inhibition of histamine H1/H4 receptors and histidine decarboxylase	Anti-allergic / anti-inflammatory effect	In vitro (RBL-2H3 cells)	Do et al. (2021a)
Camu-camu	INDIRECT	Regulation of NFAT and Nrf2 in high-glucose human keratinocytes	Nrf2 / anti-inflammatory effect	In vitro (keratinocytes)	Do et al. (2021b)
Camu-camu	INDIRECT	Amazonian fruits for noncommunicable diseases, including camu-camu	NCDs / therapeutic potential	Narrative review	Lima et al. (2024)
**Cocoa/cacao (Theobroma cacao)**					
Cocoa/cacao	**DIRECT**	Cocoa husk extract reduces α-synuclein aggregation and toxicity in PD models	α-synuclein / PD	In vitro + in vivo (PD models)	Tripodi et al. (2024)
Cocoa/cacao	**DIRECT**	Cocoa improves mitochondrial biogenesis through PPARγ/PGC1α in MPP+-intoxicated SH-SY5Y cells	Mitochondrial biogenesis / PD model	In vitro (SH-SY5Y neuroblastoma + MPP+)	Chidambaram et al. (2020)
Cocoa/cacao	**DIRECT**	Neuroprotective and immunomodulatory effects of guaraná plus cocoa husk in PD-related models	Immunoprotection / PD	In vitro / review	Azzolin et al. (2025)
Cocoa/cacao	INDIRECT	Cocoa modulates the BDNF pathway in a human Alzheimer's disease model	BDNF / neuroprotection (Alzheimer's disease)	In vitro (Alzheimer's disease model)	Cimini et al. (2013)
Cocoa/cacao	INDIRECT	Cocoa flavanols improve cognitive function, blood pressure, and metabolic profile in older adults	Cognition / vascular health	RCT (older humans; CoCoA Study)	Mastroiacovo et al. (2015)
Cocoa/cacao	INDIRECT	Cocoa-rich diet potentiates BDNF expression and hippocampal neurogenesis in healthy mice	BDNF / neurogenesis	In vivo (healthy mice)	Melgar-Locatelli et al. (2024)
Cocoa/cacao	INDIRECT	Cocoa extract and inflammaging biomarkers in older adults	Inflammaging / aging	RCT (older humans; COSMOS)	Li et al. (2025)

Food	Type	Mechanism/evidence	Subtype	Model/study	Reference
Cocoa/cacao	INDIRECT	Antioxidant polyphenolic compounds isolated from Theobroma cacao	Antioxidant / composition	Analytical study	Sanbongi et al. (1998)
Cocoa/cacao	INDIRECT	Polyphenol content in cocoa beans according to variety, region, and processing	Composition / processing	Analytical review	Oracz et al. (2015)
Cocoa/cacao	INDIRECT	Effect of pulp preconditioning on polyphenol content during fermentation	Composition / technology	Experimental study	Nazaruddin et al. (2006)
**Guaraná (Paullinia cupana)**					
Guaraná	**DIRECT**	Guaraná protects SH-SY5Y dopaminergic neurons against rotenone-induced cytotoxicity	Experimental PD model (rotenone)	In vitro (SH-SY5Y + rotenone)	de Oliveira et al. (2011)
Guaraná	**DIRECT**	Neuroprotective and immunomodulatory effects of guaraná plus cocoa husk in PD-related models	Immunoprotection / PD	In vitro / review	Azzolin et al. (2025)
Guaraná	**DIRECT**	Polyphenolic extracts protect against paraquat/rotenone neurotoxicity in PD cellular models	Experimental PD model (paraquat/rotenone)	In vitro (PD cellular models)	Tambe et al. (2023)
Guaraná	INDIRECT	Neuroprotective potential of açaí and guaraná	General neuroprotection	Narrative review	Queiroz et al. (2023)
Guaraná	INDIRECT	Habitual guaraná consumption and metabolic morbidities in older Amazonian adults	Metabolic health / epidemiology	Epidemiological study (older adults)	Krewer et al. (2011)
Guaraná	INDIRECT	Anti-aging and antioxidant potential of roasted guaraná seeds in C. elegans	Anti-aging / antioxidant effect	In vivo (C. elegans)	Peixoto et al. (2017)
Guaraná	INDIRECT	Guaraná extends C. elegans lifespan through changes in bacterial metabolism	Longevity / metabolism	In vivo (C. elegans)	Reigada et al. (2022)
Guaraná	INDIRECT	Drosophila model for studying guaraná seed functions	General experimental model	In vivo (Drosophila)	Manica-Cattani et al. (2025)
Guaraná	INDIRECT	Neurocognitive and neurological effects of coffee and caffeine, in the context of methylxanthines	Caffeine / general neurology	Narrative review	Pergolizzi et al. (2025)
Guaraná	INDIRECT	Caffeine and human body function, in the context of methylxanthines	Caffeine / general health	Narrative review	Rodak et al. (2021)
Guaraná	INDIRECT	Metabolomic profile of guaraná seeds assessed by UPLC-QTOF-MSE and NMR	Chemodiversity / composition	Analytical study	de Aquino Gondim et al. (2024)
Guaraná	INDIRECT	Methylxanthine and flavonoid content of guaraná under different drying and UV techniques	Composition / technology	Analytical study	da Silva Junior et al. (2024)

Abbreviations: BBB, blood–brain barrier; BDNF, brain-derived neurotrophic factor; MCI, mild cognitive impairment; MPP+, 1-methyl-4-phenylpyridinium; PD, Parkinson's disease; RCT, randomized controlled trial.
